# Revisiting mutational resistance to ampicillin and cefotaxime in *Haemophilus influenzae*

**DOI:** 10.1186/s13073-024-01406-4

**Published:** 2024-12-04

**Authors:** Margo Diricks, Sabine Petersen, Lennart Bartels, Thiên-Trí Lâm, Heike Claus, Maria Paula Bajanca-Lavado, Susanne Hauswaldt, Ricardo Stolze, Omar Jiménez Vázquez, Christian Utpatel, Stefan Niemann, Jan Rupp, Inken Wohlers, Matthias Merker

**Affiliations:** 1 Molecular and Experimental Mycobacteriology, Research Center Borstel, Borstel, Germany; 2 German Center for Infection Research, Partner Site Hamburg-Lübeck-Borstel-Riems, Borstel-Lübeck, Germany; 3 Evolution of the Resistome, Research Center Borstel, Leibniz Lung Center, Parkallee 1, Borstel, 23845 Germany; 4 Biomolecular Data Science in Pneumology, Research Center Borstel, Borstel, Germany; 5 National Reference Centre for Meningococci and Haemophilus Influenzae (NRZMHi), Institute for Hygiene and Microbiology, University of Würzburg, Würzburg, Germany; 6 Haemophilus Influenzae Reference Laboratory, Department of Infectious Diseases, National Institute of Health Doutor Ricardo Jorge, Lisbon, Portugal; 7 Department of Infectious Diseases and Microbiology, University of Lübeck, Lübeck, Germany; 8 University of Lübeck, Lübeck, Germany

**Keywords:** *Haemophilus influenzae*, Ampicillin resistance, Cefotaxime resistance, BLNAR, MIC, Genome-wide association study, Phylogenomics, Literature review, Resistance groups, PBP3, Haplotype networks

## Abstract

**Background:**

*Haemophilus influenzae* is an opportunistic bacterial pathogen that can cause severe respiratory tract and invasive infections. The emergence of β-lactamase-negative ampicillin-resistant (BLNAR) strains and unclear correlations between genotypic (i.e., gBLNAR) and phenotypic resistance are challenging empirical treatments and patient management. Thus, we sought to revisit molecular resistance mechanisms and to identify new resistance determinants of *H. influenzae*.

**Methods:**

We performed a systematic meta-analysis of *H. influenzae* isolates (*n* = 291) to quantify the association of phenotypic ampicillin and cefotaxime resistance with previously defined resistance groups, i.e., specific substitution patterns of the penicillin binding protein PBP3, encoded by *ftsI*. Using phylogenomics and a genome-wide association study (GWAS), we investigated evolutionary trajectories and novel resistance determinants in a public global cohort (*n* = 555) and a new clinical cohort from three European centers (*n* = 298), respectively.

**Results:**

Our meta-analysis confirmed that PBP3 group II- and group III-related isolates were significantly associated with phenotypic resistance to ampicillin (*p* < 0.001), while only group III-related isolates were associated with resistance to cefotaxime (*p* = 0.02). The vast majority of *H. influenzae* isolates not classified into a PBP3 resistance group were ampicillin and cefotaxime susceptible. However, particularly group II isolates had low specificities (< 16%) to rule in ampicillin resistance due to clinical breakpoints classifying many of them as phenotypically susceptible. We found indications for positive selection of multiple PBP3 substitutions, which evolved independently and often step-wise in different phylogenetic clades. Beyond *ftsI*, other possible candidate genes (e.g., *oppA, ridA,* and* ompP2*) were moderately associated with ampicillin resistance in the GWAS. The PBP3 substitutions M377I, A502V, N526K, V547I, and N569S were most strongly related to ampicillin resistance and occurred in combination in the most prevalent resistant haplotype H1 in our clinical cohort.

**Conclusions:**

Gradient agar diffusion strips and broth microdilution assays do not consistently classify isolates from PBP3 groups as phenotypically resistant*.* Consequently, when the minimum inhibitory concentration is close to the clinical breakpoints, and genotypic data is available, PBP3 resistance groups should be prioritized over susceptible phenotypic results for ampicillin. The implications on treatment outcome and bacterial fitness of other extended PBP3 substitution patterns and novel candidate genes need to be determined.

**Supplementary Information:**

The online version contains supplementary material available at 10.1186/s13073-024-01406-4.

## Background

*Haemophilus influenzae* is an opportunistic bacterial pathogen that can cause severe respiratory tract infections, as well as invasive infections such as septicemia, and meningitis, especially in susceptible populations such as infants, elderly adults, and immunocompromised individuals [1]. The species *H. influenzae* comprises both strains with and without a polysaccharide capsule, referred to as typeable (encapsulated) and non-typeable *H. influenzae* (NTHi) strains, respectively. Encapsulated strains are further divided into six serotypes (Hia to Hif) depending on the sequence and/or antigenicity [2].

*H. influenzae* infections used to be treated mainly with the β-lactam antibiotics ampicillin or amoxicillin. However, due to the increasing incidence of ampicillin-resistant *H. influenzae* in many countries [3–7], treatment recommendations have shifted toward β-lactam-β-lactamase inhibitor combinations or a third-generation cephalosporin. In case of known resistance or patient intolerance against these drugs, *H. influenzae* could be treated with co-trimoxazole, macrolides, fluoroquinolones, or tetracycline [1, 8, 9].

The development of resistance to ampicillin in *H. influenzae* is mainly associated with the acquisition of a β‑lactamase (TEM-1 or ROB-1) that inactivates the antibiotic, and/or alterations of the drug target, particularly the penicillin-binding protein 3 (PBP3) encoded by the *ftsI* gene, leading to a reduced affinity for β-lactams [8]. The predominant β-lactamases confer high-level resistance to penicillin and early cephalosporins, while mutational resistance through PBP3 substitutions can confer low to moderate cross-resistance against both ampicillin and third-generation cephalosporins [10–12].

To distinguish between the resistance mechanisms, *H. influenzae* strains were differentiated into (i) β-lactamase-positive ampicillin-resistant strains (BLPAR) strains, (ii) β-lactamase-negative ampicillin-resistant (BLNAR) strains, and (iii) β-lactamase-positive amoxicillin/clavulanic acid-resistant (BLPACR) strains. BLPACR are strains that are still resistant to amoxicillin, even in the presence of a β-lactamase inhibitor (clavulanic acid), indicating the presence of both resistance mechanisms [13]. Initially, strains were classified into these groups based on phenotypic tests, e.g., a chromogenic test to detect β-lactamase activity and antimicrobial susceptibility tests (ASTs) in combination with interpretative breakpoint criteria to convert minimum inhibitory concentrations (MICs) or disk diffusion zones into categories. Both the European Committee on Antimicrobial Susceptibility Testing (EUCAST) and the Clinical and Laboratory Standards Institute (CLSI) endorsed distinct AST media and different clinical breakpoints to classify *H. influenzae* isolates as susceptible and resistant. The CLSI further distinguishes an intermediate category (susceptible at increased drug concentration) [14–16].

Later, Ubukata and coworkers (2001) established a genotypic classification system, differentiating genotypic BLNAR (gBLNAR) isolates into three “resistance groups” (groups I, II, and III) according to distinct substitution patterns affecting the PBP3 transpeptidase domain [17]. In the following years, additional PBP3 substitutions likely related to resistance were identified and several authors extended, modified, or renamed the groups [6, 12, 18–24]. However, these studies also revealed that some gBLNAR isolates had MICs below the clinical breakpoint and thus would be classified as β-lactamase-negative ampicillin-susceptible (BLNAS) [19, 20, 25].

The lack of consensus with regard to the PBP3 group classifications, as well as discrepancies between resistance phenotypes and genotypes result in uncertainties about the molecular basis of mutational β-lactam resistance.

To better understand the link between genotypic and phenotypic ampicillin and cefotaxime resistance in *H. influenzae*, we performed three analyses: (I) a systematic literature review and meta-analysis for ampicillin and cefotaxime to show phenotype/genotype correlations of known PBP3 groups; (II) a phylogenomic analysis with a publicly available global and diverse *H. influenzae* dataset to identify new *ftsI* mutations under selection and possible clade effects; (III) a microbial genome-wide association study (GWAS) of a novel clinical cohort from three European centers to cross-validate mutations from the global dataset and to identify new determinants of ampicillin resistance.

## Methods

### *H. influenzae* datasets

In this paper, three different datasets were used to better understand the relationship between β-lactam-resistant phenotypes and genotypes (Additional file 1: Fig. S1).

First, *291* β-lactamase negative *H. influenzae* isolates that were identified in a literature review were used in a *meta-analysis* to assess the association of PBP3 groups with phenotypic resistance (Additional file 2: Table S1 and Additional file 1: Fig. S2).

Second, for the phylogenomic analysis, we used a *global set* of *555 H. influenzae* genomes deposited in the public database pubMLST (Additional file 2: Table S2 and Additional file 1: Fig. S3). On 26 September 2022, 2792 *H. influenzae* genomes were available on this website [26]. For each of these genomes, the multi-locus sequence type (ST; integer corresponding to a unique combination of the loci *adk*, *atpG*, *frdB*, *mdh*, *pgi*, and *recA*) and the allele number of the full *ftsI* gene (defined as loci HAEM1263 in the *H. influenzae* pubMLST scheme) were extracted from pubMLST. To reduce tree size and exclude bias due to recent outbreaks/transmission events, we only included one isolate per unique combination of STs and HAEM1263. This resulted in a high-quality phylogenetically diverse set of 555 genomes collected in 21 countries between 1944 and 2022, including both β-lactamase negative and β-lactamase positive isolates. Phenotypic AST data were not available for most isolates in this cohort.

Finally, we performed whole-genome sequencing of 322 β-lactamase negative *H. influenzae* isolates collected during routine diagnostics between 2015 and 2021. These isolates, for which phenotypic data was already available, were sourced from three European centers: the University Clinic Schleswig–Holstein in Lübeck, Germany, the National Reference Center for Meningococci and *H. influenzae* (NRZMHi) in Würzburg, Germany, and the *Haemophilus influenzae* Reference Laboratory in Lisbon, Portugal (Additional file 1: Fig. S4). If the pairwise distance between strains was fewer than ten single-nucleotide variations (SNV) [27], only one strain was retained to eliminate potential bias arising from clonality. The resulting *clinical cohort* that was used for the GWAS and comprised *298* isolates from 298 different patients (Additional file 2: Table S3).

### Systematic literature review and meta-analysis

A systematic literature review was conducted to summarize known associations of mutations in the transpeptidase domain of *ftsI* with phenotypic β-lactam resistance in β-lactamase-negative *H. influenzae* isolates (Additional file 1: Fig. S2). We searched PubMed with information available until 11th of January 2023 using the following search terms: “*ftsI* AND (mutation OR amino acid substitution) AND BLNAR [Title/Abstract]”. Filters were applied to exclude reports written in languages other than English or German and reports without full-text availability. Two papers [23, 28] published by our collaborators were added. Studies were included that fulfilled the following criteria: (1) report of MIC values for ampicillin and/or cefotaxime for individual BLNAR *H. influenzae* isolates, (2) use of gradient diffusion strips (e.g., Etest by bioMérieux/AB Biodisk, E-strip by Oxoid) or broth microdilution for MIC determination, (3) DNA (or amino acid (AA)) sequence data available for mutations in the *ftsI* region encoding the transpeptidase domain of PBP3, and (4) inclusion of at least five β-lactamase negative isolates.

The following information was extracted from each eligible study: title, last name of first author, month and year of publication, method used for MIC determination, region of *ftsI* screened for mutations, country and year of isolation of clinical isolates, accession numbers, isolate IDs (if not available, isolate IDs were generated from last name of first author and ascending numbers) with the respective information on presence/absence of β-lactamases, MICs of β-lactam antibiotics, and AA substitutions in PBP3 within the *ftsI* codon 350 to 530. Unless otherwise stated by the authors, listed AA substitutions were presumed to be the only detected AA substitutions within the specified region of PBP3. Isolates not classified into one of the previously defined PBP3 groups (I, II, or III related) implicated in resistance to ampicillin and/or cephalosporines are referred to as “genotypic wild type” in the context of this manuscript.

Odds ratios (OR) with 95% confidence intervals (95% CI) were calculated for PBP3 groups according to [29] with the following formula:

$$\text{OR}=\frac{a/b}{c/d}$$

$$95\% CI={e}^{\left(\text{ln}\left(\text{OR}\right)\pm 1.96\bullet \sqrt{\frac{1}{a}+\frac{1}{b}+\frac{1}{c}+\frac{1}{d}}\right)}$$

Here, *a* is the amount of resistant isolates classified as the PBP3 group to be analyzed, *b* the amount of resistant isolates lacking any reported substitution between AA 350 and 530, and isolates with substitution patterns different from any PBP3 group, *c* is the amount of susceptible isolates classified as the PBP3 group to be investigated, and d the amount of susceptible isolates lacking any reported substitution between AA 350 and 530, and isolates with substitution patterns different from any PBP3 group. Sensitivity was calculated with *a*/(*a* + *b*) and specificity was calculated with *d*/(*d* + *c*).

Isolates tested with gradient diffusion strips and broth microdilution using EUCAST-recommended media were classified as resistant according to EUCAST clinical breakpoints (MIC_AMPICILLIN_ > 1 mg/L, MIC_CEFOTAXIME_ > 0.125 mg/L). Isolates tested with broth microdilution and CLSI-recommended media were classified as resistant according to CLSI clinical breakpoints with intermediate isolates categorized as resistant (MIC_AMPICILLIN_ > 1 mg/L, MIC_CEFOTAXIME_ > 2 mg/L). Isolates that were phenotypically analyzed with more than one method and resulted in ambiguous discrimination of resistance and susceptibility were excluded. For contingency tables with zero counts, we added 0.5 to each cell [30]. *Z*-test was performed to assess the significance of odds ratios. *P*-values less than 0.05 were considered statistically significant. Bar plots visualizing MIC distributions in doubling dilutions for ampicillin and cefotaxime were plotted with the ggplot package v3.4.4. To visualize individual PBP3 AA substitutions together with ampicillin and cefotaxime resistance, circos plots were created using the circlize package v.0.4.15.

### Population genomic analysis of *ftsI* mutations in a global cohort of *H. influenzae* isolates

We determined the core genome of the 555 isolates from the global cohort (see above) using the pubMLST/bigsDB genome comparator [26] with *H. influenzae* serotype b strain 10,810 (NC_016809.1 with 1928 coding regions) as reference genome. The resulting core genome (loci present in at least 95% of the isolates) consisted of 1429 loci. The serotype (metadata column “genotype”) as well as the allele numbers for *H. influenzae* loci HAEM1263 (covering the full *ftsI* sequence) and *ftsI* (covering part of the *ftsI* sequence corresponding to the PBP3 transpeptidase domain AA 326–532) were retrieved from pubMLST using the “export dataset” function.

### Homoplasy analysis of *ftsI* mutations

For the identification of *ftsI* sites possibly under positive selection and thus possibly contributing to resistance, we used HomoplasyFinder as described previously [31]. Briefly, the tool detects SNPs that occur independently at the tips of a phylogenetic tree, i.e., in samples that do not share a direct common ancestor.

### DNA extraction, serotyping, and phenotypic antimicrobial susceptibility testing of the clinical cohort

Species were determined using MALDI-TOF (Lübeck), by phenotypic characterization, detection of the *H. influenzae*-specific genes *fucK* and *ompP2* and, if necessary, *ompP6*-sequencing (Würzburg) and Gram staining and MALDI-TOF mass spectrometry (Bruker, Germany) (Portugal). Total genomic DNA was extracted from fresh cultures with Qiagen Genomic-tip 100/G isolation kit (Lübeck), ANDiS automated DNA extraction, or NucliSens easyMAG platform (bioMérieux), according to the manufacturer’s instructions (Portugal) or Promega Wizard Genomic DNA Purification Kit (Würzburg), according to the manufacturer’s instructions.

For the Portuguese samples, antimicrobial susceptibility testing was performed by a broth microdilution method with commercially available minimum inhibitory concentration (MIC) panels (MiCroSTREP plus; Beckman Coulter Inc., USA), following the EUCAST guidelines and interpreted using the established European Committee on Antimicrobial Susceptibility Testing (EUCAST, v11.0 [32]) clinical breakpoints for *H. influenzae*. β-Lactamase production was detected by the chromogenic cephalosporin assay, using nitrocefin as β-lactamase substrate (Oxoid Limited, Hampshire, UK). ATCC10211 (BLNAS), ATCC49247 (BLNAR), and NCTC11315 (BLPAR) were used as control strains for susceptibility testing.

For the German samples, antimicrobial susceptibility tests were performed using benzylpenicillin disks (Penicillin PG 1 by MAST Diagnostica, Reinfeld, Germany) and minimal inhibitory concentration (MIC) test strips (from biomerieux (Etest) or Liofilchem) following the application guide provided by the manufacturer. All results were interpreted using the guidelines and clinical breakpoints of the European Committee on Antimicrobial Susceptibility Testing (EUCAST, v11.0 [32]). BBL Cefinase™ discs (Becton, Dickinson and Company, USA) were used for the detection of β-lactamase activity.

### Whole-genome sequencing of the clinical cohort

Short-read DNA libraries for the 322 clinical isolates were prepared from extracted genomic DNA with a modified Illumina Nextera XT library kit protocol as described previously [33]. Libraries were sequenced with 150 bp paired-end reads on an Illumina NextSeq 500 or 2000 instrument (Illumina, San Diego, CA, USA). Short-read data (fastq files) are available in NCBI under the Sequence Read Archive (SRA), with BioProject ID PRJEB72440 and PRJEB26586. Individual run accession numbers and biosample IDs are provided in Additional file 2: Table S3. For all clinical isolates, serotype, species, multi-locus sequence type (ST), and presence of β-lactams were re-evaluated with the whole-genome sequencing-based bioinformatic pipeline HaemoSeq as described earlier [34] (https://github.com/ngs-fzb/HaemoSeq).

In addition, we assembled a new reference genome (BioSampleID SAMN39831887) for the type strain Rd KW20 (ATCC 51907) from the DSMZ German Collection of Microorganisms and Cell Cultures GmbH, Münster, Germany (DSM-11121), employing the single-molecule real-time (SMRT), long read technology of the Sequel II system (Pacific Biosciences, CA, USA). This was required as the original complete genome of Rd KW20 (NC_000907.1) has been suppressed from RefSeq due to quality issues (too many frameshifted proteins). The DNA library was prepared with the SMRTbell Express Template Prep Kit 2.0 with barcoded adapters from IDT (Integrated DNA Technologies, USA). De-novo genome assembly was performed using the PacBio SMRTlink software v9.0 and its “Microbial Assembly” application, with the genome length set to 1.8 Mb and a seed coverage of 30. The assembly of 45,664 CCS ≥ Q20 reads (N50 CCS read length = 10,950 bp, 249 × mean coverage) resulted in one closed contiguous sequence (contig) of 1,830,702 bp. The contig was annotated with Bakta v1.8.2 [35], and three insertions (affecting an intergenic region, *hsdM*, and *lptB*) as well as one deletion (affecting *pyrG*) were curated based on an Illumina reference mapping.

Illumina short-read sequencing data were mapped to this novel curated reference genome with the MTBseq pipeline [36], which includes Burrows-Wheeler Aligner Alignment (BWA) mapping [37] and allele calling with the Genome Analysis Toolkit (GATK) [38]. Mutations (SNV) and short insertions and deletions (indels) were called with the following thresholds: four reads mapped in both forward and reverse orientation, a minimum of eight reads with a PHRED score ≥ 20 and ≥ 75% allele frequency. A summary of variant calls is provided in an HTML-based interactive figure (Additional file 3: Material S1) that gives isolate mouse-over information. All samples had a minimum average genome-wide coverage of 88 × , and at least 89% of the reads covering the reference sequence (Additional file 2: Table S3). For subsequent phylogenetic analysis, SNVs were concatenated when at least 95% of all isolates fulfilled the abovementioned thresholds for read coverage, PHRED score, and frequency at individual genome positions.

### Phylogenetic analysis

Fasttree v.2.1.9 was used with a GTR + gamma substitution model and 500 bootstrap replicates to generate a phylogenetic tree of the global and clinical cohort. Figtree v.1.4.4 was used to root this tree at midpoint and iTol v.6.8.1 was used for the visualization of mutation patterns, resistance groups, and serotypes.

### Microbial GWAS and fine-mapping of the *ftsI* locus

MTBseq-generated VCF files from each newly sequenced isolate were merged into a single joint VCF file using bcftools merge. To properly address multi-allelic variants in our analysis, we split multi-allelic calls into several biallelic variants using the bcftools norm subcommand. Principal component analysis based on genome-wide variants is provided in an HTML-based interactive figure (Additional file 3: Material S2), giving isolate information at mouse-over. Microbial genome-wide association study (GWAS) was used to test the association with resistance status (i.e., susceptible or resistant) based on a logistic regression model. Associations with MIC measurements were tested in a second GWAS, in which linear regression was applied using log2-normalized MIC values as phenotype. Two MIC measurements specified as “ > 8” were set to 8. Variants observed in fewer than 10 isolates were discarded in both settings to avoid spurious associations. Overall, 142,242 variants were tested in MIC GWAS and 152,459 variants in resistance status GWAS. Using the lm function within R (v4.3.1), the linear model was fitted for each variant as the independent variable and the log2-transformed MIC value as the dependent variable, providing *p*-values and estimated effect sizes. Accordingly, the logistic regression was fitted to the binary resistance status using the glm function, which provides *p*-values and odds ratios.

Additionally, linear and logistic regression were performed using all *ftsI* variants observed in more than 10 isolates within a joint model for resistance status (resistant or susceptible) and MIC values, respectively. Model performance as well as the significance of individual variants within the model was assessed.

Combinations of *ftsI* variants, i.e., haplotypes, were computed using an R-based interactive Shiny application (https://github.com/ricardoStolze/PegasShiny; commit 41ef697). This application internally utilizes the pegas R package for network analyses and visualization [39]. The networks presented in this manuscript utilize the TCS algorithm [40] as implemented in the pegas function “haploNet”.

### Variant and GWAS data processing and visualization

Data processing and visualization were performed in Python (v3.10.3). Loading of VCF files and variant filtering were carried out with sgkit (v0.7.0, https://github.com/sgkit-dev/sgkit) [41]. Pandas (v2.1.2, https://github.com/pandas-dev/pandas) was used to read phenotype files and to generate Additional file 2: Tables. Interactive HTML-based figures were generated with plotly (v5.17.0, https://github.com/plotly/plotly.py), except for PCA plots, which were created with bokeh (v.3.3.0, https://github.com/bokeh/bokeh). Linkage disequilibrium computations were performed using PLINK (v1.9, https://github.com/chrchang/plink-ng) [42]. The workflow and source code are available at Github (https://github.com/lbartels7/hinf).

## Results

### Associations of known *ftsI* mutations and resistance groups with phenotypic resistance

We performed a systematic literature review and meta-analysis to correlate ampicillin and cefotaxime resistance with existing PBP3 group definitions. Overall, 46 studies were eligible for screening the full text, of which 34 (74%) did not meet the eligibility criteria, mainly due to the presentation of collated/pooled data only, or the absence of DNA sequencing data. One additional study was excluded because of nomenclature conflicts of reported mutations (Additional file 1: Fig. S2, Additional file 2: Table S1).

In total, we included genotypic and phenotypic data from 12 studies on 291 β-lactamase negative *H. influenzae* isolates in the meta-analysis. Quantitative drug susceptibility testing (MIC determination) was performed by gradient diffusion strips (e.g., Etest by bioMérieux/AB Biodisk, E-strip by Oxoid) and interpreted using EUCAST clinical breakpoints (*n* = 167 isolates; 57.47%) as well as broth microdilution (BMD) with CLSI recommended medium and clinical breakpoints (*n* = 124 isolates; 42.6%) and BMD with EUCAST recommended medium and clinical breakpoints (*n* = 63 isolates; 21.6%), respectively. Ampicillin MIC values determined by at least one of the above methods were available for 284/291 isolates (97.6%), and cefotaxime MIC values were available for 134/291 isolates (46.0%). Of those 146/284 (51.4%) were resistant to ampicillin according to EUCAST (or at least intermediate according to CLSI) (MIC > 1 mg/L). Concerning cefotaxime resistance, 20/127 (15.7%) isolates tested with gradient diffusion strips and broth microdilution using EUCAST recommended media were resistant (MIC > 0.125 mg/L), whereas all 7 isolates tested with broth microdilution using CLSI recommended media and clinical breakpoints were susceptible (MIC ≤ 2 µg/mL). Data for other β-lactams were scarce and therefore not used for further analysis.

**Fig. 1:**
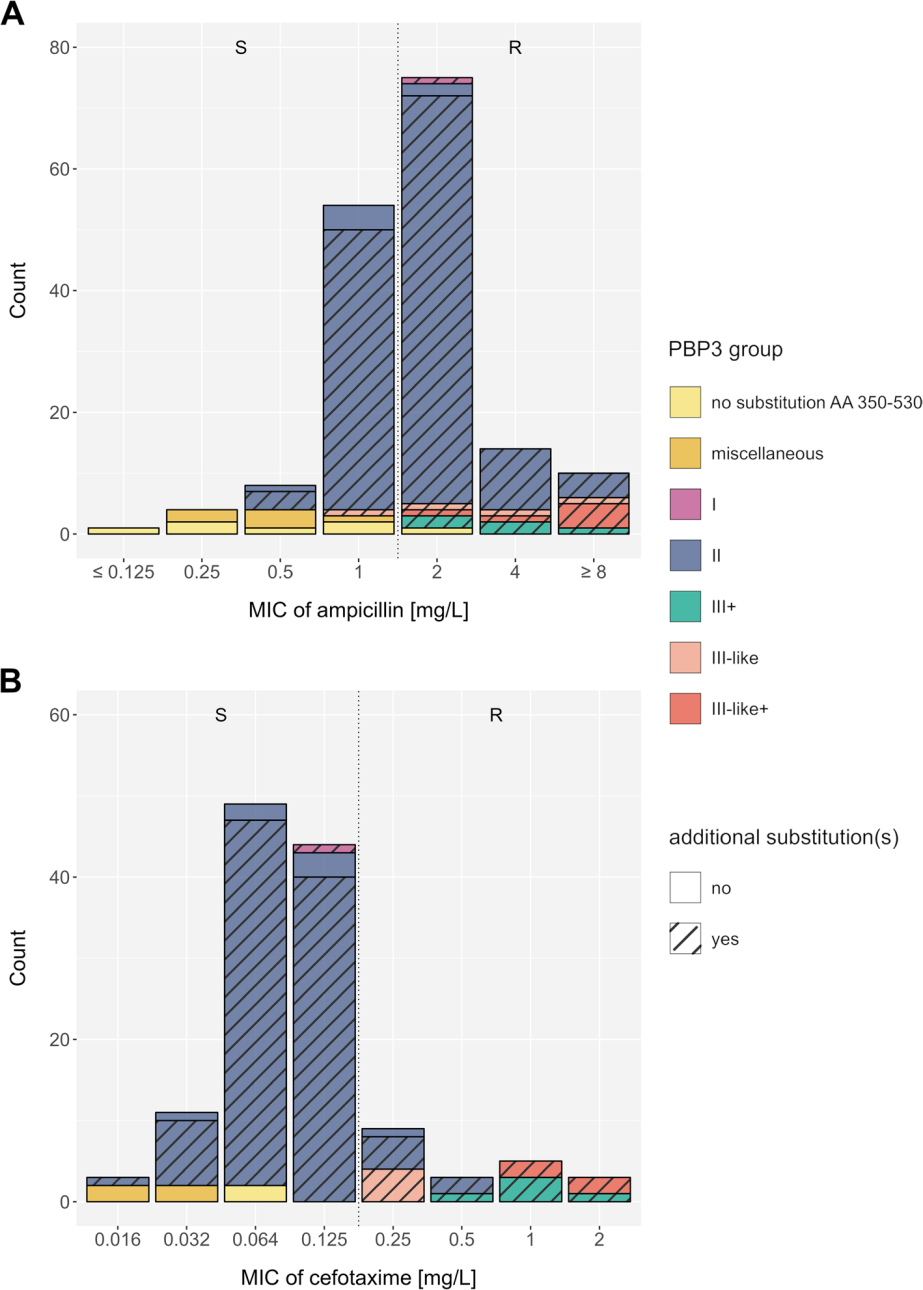
Distribution of minimum inhibitory concentrations (based on gradient diffusion strips (e.g., Etest)) for **A** ampicillin (*n* = 167) and **B** cefotaxime (*n* = 127) among previously published β-lactamase negative* H. influenzae* isolates. The color code represents the main PBP3 groups, isolates with no reported substitutions within the PBP3 transpeptidase domain AA 350–530 (compared to the reference strain Rd KW20), and isolates with miscellaneous PBP3 transpeptidase substitution patterns (not matching the minimum set of mutations used to describe known groups). Hatched bars display isolates exhibiting additional PBP3 substitutions at position 350 to 530, other than the respective group-specific substitutions. Dotted vertical lines indicate clinical breakpoints (EUCAST) that differentiate phenotypically susceptible (S) and phenotypically resistant (R) isolates

For each isolate, we converted the reported mutation pattern into a PBP3 group according to the consensus nomenclature proposed by Nürnberg et al. (Table 1) [23]; other nomenclatures that have previously been used are listed in Additional file 2: Table S4. The distribution of PBP3 groups was as follows: group I (*n* = 6), group II (*n* = 235), group III + (*n* = 13), group III-like (*n* = 8), and group III-like+ (*n* = 8), while no isolate belonged to group III. Most of the isolates classified into the resistance groups also harbored additional mutations within the transpeptidase domain (Fig. 1, Additional file 1: Fig. S5 and S6). The vast majority of *H. influenzae* isolates lacking any reported PBP3 substitution in the analyzed transpeptidase domain, or not matching any of the proposed PBP3 groups were ampicillin (19/21) and cefotaxime susceptible (6/6) (Table 1, Additional file 2: Table S5). All PBP3 groups and subgroups with sufficient data (*n* > 5) were significantly associated with ampicillin resistance (*p* < 0.05), except group IIa, which was borderline significant (*p* = 0.06) (Table 1). For group III sensu stricto and group I, we could only identify < 6 isolates, and statistical significance was not assessed. For cefotaxime, only group III-related isolates were significantly associated with cefotaxime resistance (*p* = 0.02, Additional file 2: Table S5); individual subgroups could not be assessed due to the low sample size. For both ampicillin and cefotaxime, the reported associations remained robust for MICs determined with gradient diffusion test only (data not shown). Overall, MIC differences and categorical classifications into resistant and susceptible between gradient diffusion test and broth microdilution were only deemed marginally different for our cohorts (Additional file 1: Fig. S7). Notably, many group II isolates were still tested phenotypically susceptible to ampicillin with both, gradient diffusion test and broth microdilution (Fig. 1, Additional file 1: Fig. S5, S6, and S7), and thus specificities to predict resistance were low (15.6%) (Table 1). Further stratification within group II could also not resolve these discordances (Additional file 1: Fig. S8). In fact, the EUCAST breakpoint for ampicillin at 1 mg/L divides group II isolates into a resistant and a susceptible population (Fig. 1A). With regard to cefotaxime, the EUCAST breakpoint at 0.125 mg/L classified the majority of group II isolates, tested using gradient diffusion strips (100/110, 90.9%), as susceptible (Fig. 1B). Individual mutations alone were poor predictors of an ampicillin- and cefotaxime-resistant phenotype, most of which occurred in both susceptible and resistant isolates. The PBP3 substitution L389F was always related with phenotypic resistance to ampicillin and cefotaxime in tests interpreted with EUCAST breakpoints (Additional file 1: Fig. S9A-D).

**Table 1:** Associations of previously proposed PBP3 groups and ampicillin resistance for 276 β-lactamase negative *H. influenzae* isolates with stated ampicillin MIC values

PBP3 group	Amino acid substitutions							Present in AMP-resistant* strains	Absent^1^ in AMP-resistant* strains	Present in AMP-susceptible strains	Absent^1^ in AMP-susceptible strains	Odds ratio (95%CI)	*p*-value	Specificity [%]	Sensitivity [%]
	M377	S385	L389	I449	A502	R517	N526								
I [9]						R517H		2	2	3	19	6.3 (0.6–63.6)	NA	86.4	50.0
II^2^ [9]								122	2	103	19	11.3 (2.6–49.5)	*0.00135*	15.6	98.4
IIa [11]							N526K	16	2	34	19	4.5 (0.9–21.6)	0.06214	35.8	88.9
IIb [11]					A502V		N526K	61	2	40	19	14.5 (3.2–65.6)	*0.00052*	32.2	96.8
IIc [11]					A502T		N526K	39	2	20	19	18.5 (3.9–87.6)	*0.00023*	48.7	95.1
IId [11]				I449V			N526K	6	2	9	19	6.3 (1.1–37.8)	*0.04281*	67.9	75.0
III related^3^								20	2	5	19	38 (6.6–220)	*0.00005*	79.2	90.9
III [10]	M377I	S385T					N526K	0	2	0	19	#	NA	#	#
III+ [14]	M377I	S385T	L389F				N526K	8	2	1	19	76 (6–962.4)	*0.00083*	95.0	80.0
III-like [12]	M377I	S385T				R517H		4	2	4	19	9.5 (1.3–71)	*0.02821*	82.6	66.7
III-like+ [14]	M377I	S385T	L389F			R517H		8	2	0	19	132.6 (5.7–3068.1)	*0.00229*	100.0	80.0
Cumulative								144	2	111	19	12.3 (2.8–54)	*0.00087*	14.6	98.6

^*^Classification of resistant isolates by gradient diffusion strips and broth microdilution with EUCAST recommended media was done according to EUCAST clinical breakpoint (MIC > 1 mg/L). Classification of resistant isolates by broth microdilution with CLSI recommended media was done according to CLSI clinical breakpoints with intermediate treated as resistant (MIC > 1 mg/L). Strains (*n* = 8) that were phenotypically analyzed by more than one method and led to an ambiguous classification were excluded

^1^Includes isolates lacking any reported amino acid substitution between codon 350 and 530, and substitution patterns different from any PBP3 group

^2^All group II isolates irrespective of the group II subgroups

^3^All group III+ , III-like and III-like+ isolates

^#^No data available for this group, *p*-values < 0.05 marked in italics, NA *p*-value not calculated for groups with < 6 isolates

### Population genomic analysis of PBP3 mutations and groups in a global *H. influenzae* cohort

Next, we sought to investigate whether some of the previously proposed PBP3 resistance-associated groups might be phylogenetically informative, i.e., whether isolates belonging to the same PBP3 group also cluster together in a global phylogeny. To this end, we used a population genomic approach with a global collection of 555 *H. influenzae* isolates deposited in the pubMLST database [26]. The collection included a diverse non-redundant set of isolates from 21 countries (1944–2022) (see “ Methods”). The isolates belong to 414 different STs and represent 287 different HAEM1263 alleles (loci in pubMLST corresponding to full-length *ftsI*) and 158 *ftsI* alleles (loci in pubMLST corresponding to the PBP3 transpeptidase domain (AA 326–532)) (Additional file 2: Table S2).

**Fig. 2:**
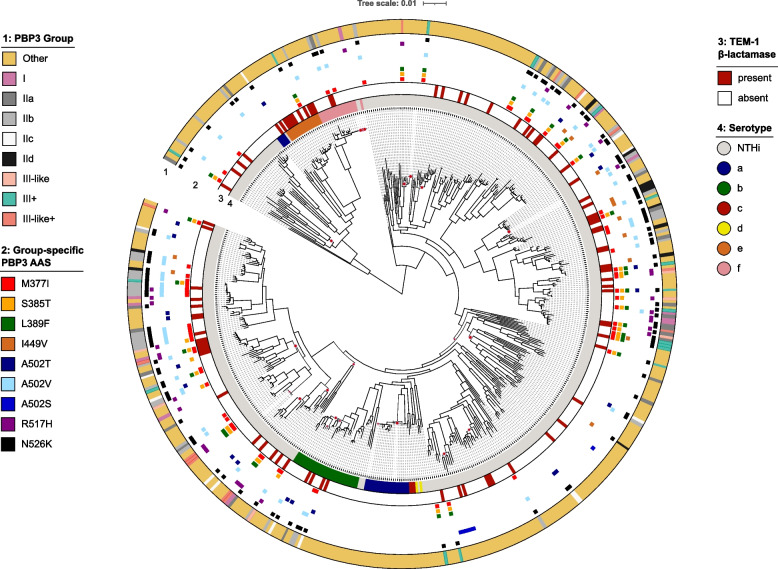
Phylogeny of a global and diverse collection of 555 *H. influenzae* isolates based on a core-genome alignment of 1429 genes. The category “other” represents all genomes without a PBP3 group-specific substitution or with a PBP3 substitution at position 502 only. Branches that have a local support value of less than 0.8 are highlighted with red dots. AAS amino acid substitutions in PBP3, NTHi non-typeable *H. influenzae*

Overall, 149/555 isolates (26.8%) were classified into one of the previously described resistance groups, 394/555 isolates (71.0%) had none of the group-specific substitutions, and 12/555 isolates (2.1%) had either only A502T, A502S, or A502V, respectively (Additional file 2: Table S2). No group III representative substitution pattern, sensu stricto, was identified. Interestingly, neither of the serotype a, d, or c isolates had group-specific mutations. Out of 149 isolates classified in one of the resistance groups, 42 (28.2%) had additional group-specific mutations aside from the minimum set defining the group (Table 1). For instance, within group IIb (A502V + N526K), the majority (35/53, 66%) also had the additional substitution M377I, which is characteristic of group III strains. The global phylogeny of *H. influenzae* (Fig. 2) shows that group-specific resistance mutations and combinations are not phylogenetically informative as they are distributed across independent lineages (i.e., sequence types).

**Fig. 3:**
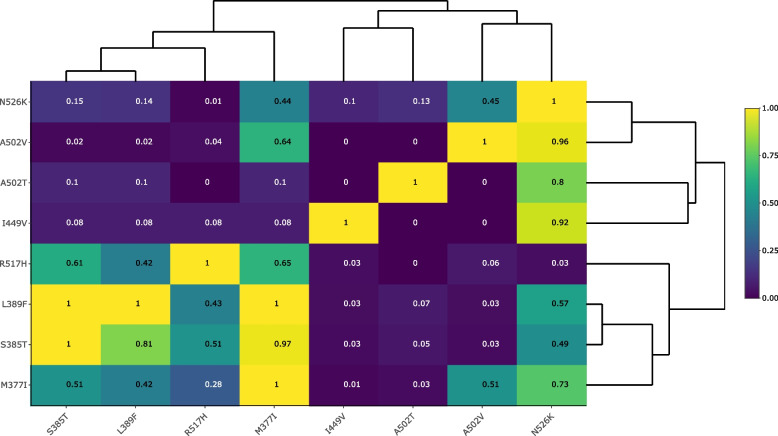
Co-occurrence of PBP3 substitutions in a global *H. influenzae* collection (*n* = 555). Hierarchical clustering based on proportions of PBP3 group-specific substitutions on the* y*-axis that co-occurred with PBP3 substitutions on the *x*-axis

Interestingly, N526K and R517H rarely occurred together in one isolate (Fig. 3). On the other hand, we confirmed the genomic linkage of group III mutations, i.e., L389F, S385T, and M377I. Specifically, the mutation L389F always co-occurred with the group mutations M377I and S385T in our global dataset (Fig. 3), and L389F mostly co-occurred with D350N and S357N, two other putative resistance-related substitutions (Additional file 2: Tables S2 and S4). Overall, the pairwise comparison of all group-specific substitutions revealed very common substitution patterns as well as combinations that never co-existed in one isolate (Fig. 3).

Next, we performed a homoplasy analysis of the full *ftsI* gene in order to identify additional mutations possibly under positive selection. Notably, this analysis might also highlight alleles under relaxed purifying selection that have no measurable effect on the bacterial fitness. Overall, we identified 272 mutations showing signs of homoplasy, i.e., identical mutations in phylogenetically unrelated subgroups. Plotting the top 100 hits from the homoplasy analysis, as judged by the lowest consistency indices, suggested an accumulation of mutations within the transpeptidase domain of the *ftsI* gene (Additional file 1: Fig. S10). However, we also identified numerous other mutations outside this domain in the homoplasy analysis and likely neutral synonymous mutations not affecting the protein structure (Additional file 2: Table S6). The top PBP3 substitutions from the homoplasy analysis were V547I, E603D, D350N, N569S, and P31S, respectively.

### Genome-wide variant associations with ampicillin resistance in a new clinical *H. influenzae* cohort

**Fig. 4:**
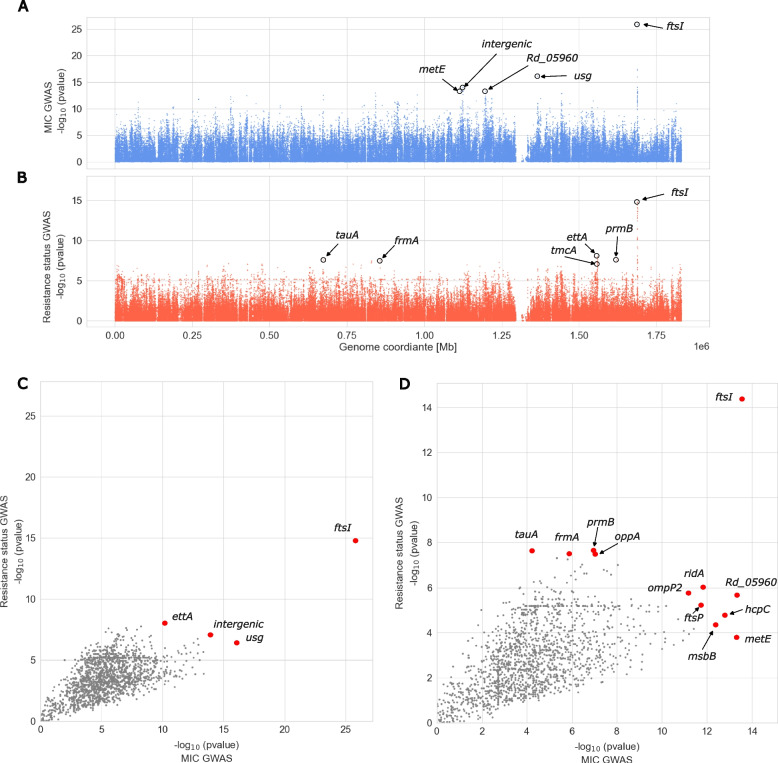
The -1 × log10 transformed *p*-values of variant associations genome-wide. In panels **A** and **B**, each dot represents a variant, in panels **C** and **D**, each dot represents a gene. **A** Manhattan plot of MIC GWAS. **B** Manhattan plot of resistance status GWAS. **C** Gene-wise most significant MIC-association *p*-value (*x*-axis) versus most significant resistance status-associated* p*-value (*y*-axis). Whereas** C** considers all variants occurring in more than 10 isolates, **D** considers thereof only the subset of variants causing amino acid substitutions. Only ftsI had in both GWASs a much smaller *p*-value compared to the remaining genes. Respective HTML-based interactive figures providing detailed information on individual data points at mouse-over are provided as Additional file 3: Material S3 (panel **A**), 4 (panel **B**), 5, (panel **C**), and 6 (panel **D**)

In order to identify additional *ftsI* mutations and genes other than *ftsI* that might be related to ampicillin resistance and to better explain the observed MIC distributions (Additional file 1: Fig. S11), we performed two microbial genome-wide association studies (GWAS) employing a novel clinical cohort. First, we used log2-transformed MIC values as phenotype (available for 247 isolates). Second, we used the binary phenotypic classification (available for 298 isolates), i.e., resistant or susceptible based on EUCAST guidelines, in the following referred to as “resistance status”. In both approaches, the only prominently associated locus was *ftsI* (Fig. 4A and B). In the GWAS using the binary classification resistant or susceptible, several *ftsI* variants had *p*-values of less than 10^–14^, whereas all other genomic variants had *p*-values greater than 10^–9^ (Fig. 4B). Likewise, in the GWAS based on MIC values, *ftsI* variants had association *p*-values less than 10^–25^ in comparison to *p*-values larger than 10^–17^ for other genome-wide variants (Fig. 4A). The most strongly associated PBP3 substitution in both GWAS approaches was M377I (*p* = 2.49 × 10^–14^ (MIC); *p* = 4.36x10^−15^ (resistance status)). We identified another four non-synonymous PBP3 substitutions that were more strongly associated with resistance than all other substitutions (*p*-value ≤ 10^–8^, Additional file 2: Table S7), namely the PBP3 group substitutions A502V and N526K as well as V547I and N569S (located outside the transpeptidase domain), in addition to several synonymous *ftsI* variants. These five are also the most strongly MIC-associated substitutions (Additional file 2: Table S7).

Next, we investigated a possible role of other genes in ampicillin resistance, by selecting the most strongly associated variant per gene from both GWAS approaches and contrasting the respective *p*-values (Fig. 4C and D)).

Except for *ftsI*, genes were preferentially associated with ampicillin resistance in only one of the two GWASs. Thus, no additional strongly supported candidate resistance gene could be identified. However, an interesting gene that came up consistently with top amino acid substitution *p*-values in both GWASs was a yet uncharacterized protein encoded by *Rd_05960* (NCBI locus tag RdKW20_001113) (Fig. 4A and D). In addition, genes *ridA* and *ompP2* rank comparably high in both GWASs. The gene *usg* encoding an oxidoreductase is the second most strongly associated gene in MIC GWAS (Fig. 4A and C) and gene *ettA* in resistance status GWAS (Fig. 4B and C), both, however, based on the presence of synonymous mutations that are typically considered non-functional. Additional genes with amino acid substitutions that are strongly associated preferentially in one of both GWASs comprise *prmB*, *tauA, oppA,* and *frmA* (GWAS based on resistance status, Fig. 4B and D) as well as *metE*, *hcpC*, *msbB,* and *ftsP* (GWAS based on MIC values, Fig. 4A and D). Finally, the intergenic variant most strongly associated with resistance status is located approximately 400 bases upstream of *hslR*, which encodes for a heat shock protein 15 homolog (Fig. 4B and C). The intergenic variant most strongly associated with MIC values is located within a cluster of molybdate ABC transportation molecules *modA*, *modB*, *modC*, and *modE*, upstream of *modB*, *modC*, and *modE* (Fig. 4A and C).

### Fine-mapping of the *ftsI* locus

We subsequently aimed to investigate how well resistance is explained by *ftsI* mutations. To this end, we used 2 joint models of 44 high-quality non-synonymous mutations in *ftsI* detected in a minimum of 10 isolates within our novel clinical cohort, 1 for resistance status, and 1 for MIC values (Additional file 2: Table S7). Surprisingly, the joint model also poorly predicted resistance status, and likewise, MIC values were poorly modeled by *ftsI* mutations (model-adjusted *R*^2^ = 0.5603). Only a few mutations significantly contributed to resistance phenotypes, nine with respect to MIC values and seven with respect to resistance status, with A502V (*p* = 0.01385 (MIC), *p* = 0.02010 (R/S)), N526K (*p* = 0.00816 (MIC), *p* = 0.06775 (R/S)), and V547I (*p* = 0.00456 (MIC), *p* = 0.00784 (R/S)), the only common significant substitutions in both models (significance level 0.1, Additional file 2: Table S7). The significant mutations from both joint models included not only the top substitutions from our GWASs, i.e., M377I, A502, V547I, N526K, and N569S, but also substitutions that are not linked to resistance when considered individually, E603D, A586S, E135Q, and N75S. Notably, N569S and V547I are in high linkage disequilibrium, a measure of nonrandom co-occurrence of alleles at two sites (squared Pearson correlation coefficient *r*^2^ = 0.71, Additional file 1: Fig. S12 and interactive HTML-based Additional file 3: Material S7) as well as the combination of M377I, A502V, and N526K (A502V/N526K *r*^2^ = 0.87; M377I/N526K *r*^2^ = 0.48; M377I/A502V *r*^2^ = 0.41, Additional file 1: Fig. S12). This was also apparent when these mutations were plotted on a phylogenetic tree: the combinations of the substitutions M377I, A502V, and N526K (i.e., group IIb-defining mutations + M377I) always co-occurred with V547I, N569S as well as with D350N/S (Additional file 1: Fig. S13). N569S always co-occurred with V547I but not vice versa. Ampicillin MICs of group IIb isolates harboring the combination M377I, A502V, and N526K (*n* = 58) were slightly higher than those for group IIb isolates without M377I (*n* = 28 with MIC available) (median 1.5 vs 1 mg/L, *p* = 0.005, Mann–Whitney *U* test). Likewise, MICs for cefotaxime were increased for PBP3 group II isolates with PBP M377I (*n* = 37) compared to group II isolates lacking the PBP M377I substitution (*n* = 18) (median 0.064 vs 0.047 mg/L, *p* = 0.028, Mann–Whitney *U* test).

Mutations in *ftsI* resulting in PBP3 group substitutions L389F and R517H were identified in less than 10 isolates of the clinical cohort and thus were not assessed via GWAS and joint model. The PBP3 group substitution I449V was not associated with resistance throughout, and S385T was only weakly associated with resistance in the MIC GWAS and joint model (*p* = 0.03210, Additional file 2: Table S7).

**Fig. 5:**
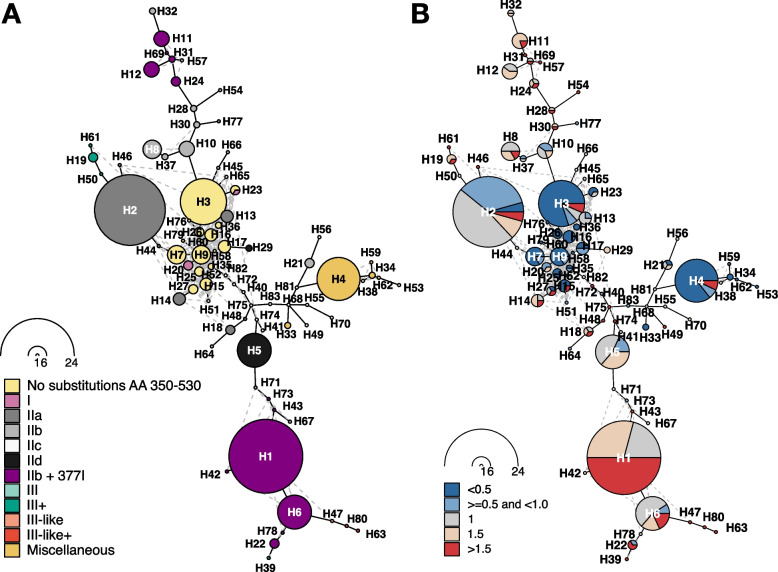
Haplotype network for 44 *ftsI *variants detected in a minimum of 10 out of overall 247 isolates. Nodes represent haplotypes, i.e., combinations of variants, and lines connect similar haplotypes. Nodes are scaled according to isolate frequency of the corresponding haplotype and haplotypes are named in the order of occurrence, starting with H1 as the most common haplotype. **A** Colors denote resistance groups and the abundant combination of group IIb variants with the additional PBP substitution M377I. **B** Colors denote minimum inhibitory concentrations (MIC) for ampicillin. Blue shades relate to isolates with MIC values considered ampicillin susceptible (MIC < 1 mg/L); red shades indicate isolates classified as ampicillin resistant (MIC > 1 mg/L). Borderline MIC values of 1 mg/L (by definition ampicillin susceptible) are indicated in gray

Finally, we investigated in the clinical cohort whether additional *ftsI* mutations or combinations thereof stratify previously established resistance groups and allow an improved prediction of phenotypic ampicillin resistance. Towards this, we constructed haplotypes representing all observed combinations composed of 44 *ftsI* mutations that are present in a minimum of 10 isolates in the clinical cohort and that cause PBP3 substitutions (12 within and 32 outside the transpeptidase domain) (Fig. 5 and Additional file 1: Fig. S14). Overall, the observed combinations resulted in 83 haplotypes, 11 of which represented more than 50% of the isolates (Additional file 2: Table S8). The most common haplotype in the clinical cohort (here denoted H1 with *n* = 24 isolates), found in both Portugal and Germany (cf. Additional file 1: Fig. S14), carried the previously mentioned combination of PBP3 substitutions M377I, A502V, and N526K, with ampicillin MICs greater than or equal to 1 mg/L (isolates from ST 1034 (*n* = 11), 14 (*n* = 12), and 1206 (*n* = 1); Additional file 2: Table S3). This combination was also present in other common haplotypes (H6 with 11 isolates from ST 367 (*n* = 8), 142 (*n* = 1), 203 (*n* = 1), and 136 (*n* = 1); H11 with 5 isolates from ST 834 (*n* = 3), 422 (*n* = 1), and 1881 (*n* = 1); H12 with 5 isolates from ST 203). In the entire cohort, the combination M377I, A502V, and N526K was found in 60 isolates, with only 3 isolates harboring an ampicillin MIC < 1 mg/L (Suppl. Table S3). Other haplotypes comprising mainly isolates with a MIC less than or equal to 1, i.e., susceptible isolates, are located in the center of the network from which different paths along similar haplotypes exist towards haplotypes observed mainly in resistant isolates (Fig. 5B)). Haplotypes of isolates without any PBP3 transpeptidase domain substitution as well as haplotypes of isolates that carry transpeptidase domain substitutions that do not belong to a currently defined PBP3 group are largely phenotypically susceptibility (Fig. 5). Specifically, out of 131 isolates without group-specific mutations or without any non-synonymous mutations between AA 350–530, 127 (96.9%) were susceptible. The four resistant ones (all with MIC = 4) belong to serotype a, serotype b, serotype f, and NTHi, respectively, and three carry a V547I mutation (Additional file 1: Fig. S14 and Additional file 2: Table S3).

## Discussion

This study aimed to better understand the relation between phenotypic and genotypic β-lactam resistance in β-lactamase negative *H. influenzae* strains. We revisited PBP3 mutations and combinations implicated in resistance to ampicillin and cefotaxime and assessed their predictive power. Although previously defined PBP3 groups were significantly associated with ampicillin resistance and not phylogenetically informative, they could not explain the full phenotypic spectrum of phenotypically susceptible and resistant isolates. We also showed associations of ampicillin resistance with PBP3 substitutions outside the transpeptidase domain and identified putative novel candidate resistance genes. However, the impact of these genes and substitutions on drug resistance, especially to cefotaxime and other β-lactam antibiotics, treatment outcome, and compensatory effects needs to be further investigated.

*H. influenzae* strains classified into one of the previously defined PBP3 resistance groups (groups I, II, and III related) often harbor MICs below the resistance breakpoint [43–46]. These inconsistencies between genotypes and phenotypes were seen with both broth microdilution and gradient diffusion strip phenotypic assays and created uncertainty with regard to treatment decisions. We show that PBP3 group II isolates are significantly associated with ampicillin resistance, but the classification of an isolate as PBP3 group II indeed has a low specificity (< 16%) to rule in resistance to ampicillin as almost half of them are classified as phenotypically susceptible. Most phenotypically susceptible PBP3 group isolates had MICs equal to the currently endorsed EUCAST clinical breakpoint of 1 mg/L for ampicillin. Lowering the clinical breakpoint for ampicillin, e.g., to 0.5 mg/L, would classify borderline isolates with known PBP3 substitution patterns as resistant; however, this possibly splits the susceptible genotypic wild-type population. To address such an uncertain interpretation, one could introduce an “area of technical uncertainty” (ATU), i.e., a warning from the laboratory that the tested isolate could harbor genotypic resistance determinants, and that phenotypic AST results are not reproducible [47]. In cases where genotypic results are available to guide treatment decisions, *H. influenzae* PBP3 groups can provide a reproducible interpretation based on the presence/absence of an underlying ampicillin resistance mechanism.

For cefotaxime (and other cephalosporins), PBP3 group III specific substitutions (different combinations of PBP3 S385T, M377I, and L389F) have been associated with relevant resistance levels, which was also confirmed in our analysis [12, 23, 48]. In addition, we found PBP3 group IIb with the additional substitution PBP3 M377I to confer a MIC increase to ampicillin and cefotaxime as compared to PBP3 group II sensu stricto. Mizoguchi et al. [49] also showed that additional PBP3 mutations (outside the transpeptidase domain) gradually impact MICs to cefotaxime. We highlight the extended PBP3 pattern: group IIb with M377I, and additionally V547I and N569S, present in the most prevalent and mostly ampicillin-resistant haplotype H1 as well as in other haplotypes. Previously, this pattern (M377I, A502V, N526K, V547I, and N569S) has been linked with PBP3 type A, which is common in *H. influenzae* strains from patients with invasive disease in Europe and Canada [22]. PBP3 M377I was also identified in the GWAS as strongly associated with ampicillin resistance. In contrast, previous studies using genetically engineered strains have shown that PBP3 M377I alone did not affect phenotypic resistance to β-lactams and thus might rather have a fitness effect (compensatory mutation) [24, 50]. Likewise, we identified the combination PBP3 V547I and N569S also in phenotypically susceptible and genotypic wild-type strains, such as haplotype H4. Thus, it could also have an impact on the bacterial fitness or virulence, or it is simply a neutral combination as a result of horizontal gene transfer of adjacent resistance alleles. Yet it is unclear if pre-existing PBP3 substitutions foster the evolution of resistance to cephalosporins or increase the rate of treatment failures.

Through phylogenomic analysis of a global and diverse set of *H. influenzae* strains, we demonstrate that known resistance-related mutation patterns within the PBP3 transpeptidase domain occur independently in different phylogenetically unrelated lineages (i.e., sequence types). This indicates that these mutations are not inherited from a recent common ancestor, i.e., they are not phylogenetically informative, but rather the result of positive selection supporting their role in conferring resistance. However, whether the mutation patterns resulted from convergent evolution (i.e., independent but identical stepwise acquisition of specific mutations in different genetic backgrounds in response to β-lactams as selective pressure) or horizontal gene transfer events remains unknown.

To explore the full spectrum of mutational resistance in *H. influenzae*, we present the first GWAS results that employ quantitative MIC values. We characterized the genomic landscape and underlying phenotypes and identified novel candidate resistance genes (e.g., *Rd_05960*, *usg, ridA, oppA,* and *ompP2*) possibly contributing to or modifying the susceptibility to ampicillin. Further, the intergenic variant that was most strongly associated with a difference in MIC values may promote or mediate resistance by altering gene expression of molybdate ABC transportation molecules [51, 52]. With ABC transporter genes and the genes *ftsI*, *oppA*, and *ompP2*, we identify resistance-related genes that are likely involved in various different aspects of β-lactam resistance according to the KEGG database of manually curated pathways (https://www.kegg.jp/entry/map01501). However, we also detect a number of genes for which possible functional mechanisms by which they may promote resistance are unclear, often because there is little information available about the genes and their proteins, e.g., *Rd_05960*, *usg*, and *ridA*. Replication of the genes reported here in an independent cohort is necessary, and GWAS statistical power is expected to increase with sample size, harmonized MIC determination methods, and additional representative cohorts from geographically spread centers.

We highlighted PBP3 substitutions and substitution patterns that often occur in a specific stepwise manner (e.g., L389F, S385T, and M377I that define group III+ and group III-like+) and across unrelated branches in the *H. influenzae* phylogeny, which suggests ongoing and constrained positive selection of the *ftsI* locus. In line with previous studies [12, 50], we found that the substitutions PBP3 N526K and R517H play a significant role, likely acting as “driver” mutations that make *H. influenzae* more prone to acquire additional PBP3 substitutions associated with elevated MICs. However, PBP3 N526K and R517H almost never co-occur, likely due to structural constraints imposed by their close physical proximity within the 3D protein structure. Our joint model of all *ftsI* variants also points towards N526K, A502V, and non-resistance group variant V547I as potential drivers, since they are the only ones significantly contributing to the prediction of both resistance status (R/S) as well as MIC measurement. These mutations are also supported by GWAS, and V547I was among the top 5 substitutions of the homoplasy analysis.

A limitation of our study is the use of a reference mapping approach that masks gene content absent in the reference isolate and thus might miss important resistance determinants in clinical isolates. This limitation can be addressed in future work by structural variant identification or by improving genome sequence representation with k-mer- or pangenome-based approaches. Finally, the high degree of genetic diversification in *H. influenzae* [53] and the high diversity of *ftsI* mutations observed in our cohorts may require consideration of rare variants for the prediction of resistance. Since only variants with high allele frequencies (typically a minimum of 5%) are accessible for standard GWAS approaches applied in this work, the inclusion of rare variants would require novel methods that are currently increasingly developed and applied in human genomic studies.

Genome-wide variations, quantitative MIC measurements in *H. influenzae,* and outcome data have the potential to train machine learning-based approaches that improve diagnostics [54]. Corresponding approaches for antimicrobial resistance prediction have recently been assessed in other bacteria [55]. Our comprehensive literature review, however, shows that currently published *H. influenzae* data are limited in size and may be biased, for example, toward isolates that carry non-wild-type *ftsI* alleles. This issue needs to be addressed prior to applying more sophisticated, genome-wide approaches for the prediction of resistance.

## Conclusions

We revisit and provide novel insights into the molecular determinants of ampicillin resistance in β-lactamase negative *H. influenzae* isolates and highlight artifacts associated with currently endorsed binary clinical breakpoints for ampicillin. Current phenotypic assays cannot reproducibly classify PBP3 group II isolates with MICs around the clinical breakpoints as resistant. Thus, we recommend the implementation of an area of technical uncertainty (ATU). When the MIC is within such an ATU and genotypic ampicillin resistance determinants are detected, e.g., PBP3 groups, they can provide further guidance. Comprehensive microbial genome-wide data, quantitative MIC measurements as well as information on treatment outcome are needed for future large-scale studies that further improve mutational resistance prediction and thus clinical decision-making.

## Supplementary Information

Additional file 1. Supplementary figures. This file contains all Supplementary Figures and the figure captions. Fig. S1 Overview of the cohorts, Fig. S2 Workflow literature review, Fig. S3 Workflow global public cohort, Fig. S4 Workflow GWAS of clinical cohort, Fig. S5 MIC distributions broth microdilution and CLSI clinical breakpoints, Fig. S6 MIC distributions broth microdilution and EUCAST clinical breakpoints, Fig. S7 Ampicillin MICs of group II isolates stratified to methods and cohorts, Fig. S8 Ampicillin MICs of group II sub-groups stratified to different methods, Fig. S9 Circos plots showing the association between PBP3 substitutions and ampicillin/cefotaxime MICs, Fig. S10 Density plot showing the distribution of *ftsI* mutations, Fig. S11 Distribution of minimum inhibitory concentration based on gradient diffusion strips for the clinical cohort, Fig. S12 Heatmap visualizing the linkage disequilibrium between all amino acid changing variants within the *ftsI* gene, Fig. S13 Phylogeny of 298 clinical beta-lactamase negative *H. influenzae *isolates from three European centers (Lübeck, Würzburg and Lisbon), Fig. S14 The haplotype network displaying the 83 combinations of all 44 variants observed in gene *ftsI* in at least 10 isolates.

Additional file 2. Supplementary Tables. This file contains all Supplementary Tables. Table S1 Isolates used for the literature review, Table S2 global public pubMLST cohort, Table S3 clinical cohort, Table S4 Previously defined PBP3 resistance groups for Haemophilus influenzae, Table S5 Previously proposed PBP3 group-specific AA substitutions and cefotaxime minimum inhibitory concentrations, Table S6 Consistency index report obtained from the homoplasy analysis, Table S7 Summary of 44 *ftsI *variants resulting in PBP3 substitutions and occurring in a minimum of 10 isolates of the clinical cohort, Table S8 Haplotypes across 44 protein-altering *ftsI* variants observed within a minimum of 10 from 247 isolates.

Additional file 3. Supplementary Material. This file contains all Supplementary Material in html format and figure captions. Material S1 Interactive histogram of the distribution of the number of variants per isolate for all 322 beta-lactamase-negative isolates, Material S2 Genome-wide variant-based interactive PCA plot, Material S3: Interactive Manhattan plot of the MIC GWAS, Material S4 Interactive Manhattan plot of the resistance status GWAS, Material S5 Interactive version of Fig. 4C, Material S6 Interactive version of Fig. 4D, Material S7 Interactive heatmap visualizing the linkage disequilibrium between all amino acid changing variants within the *ftsI *gene.

## Data Availability

Sequencing data generated as part of the study is available at ENA under IDs provided in Additional file 2: Table S3, which belong to ENA BioProject IDs PRJEB72440 (https://www.ebi.ac.uk/ena/browser/view/PRJEB72440) and PRJEB26586 (https://www.ebi.ac.uk/ena/browser/view/PRJEB26586). The *H. influenzae* assembly is available under the accession PRJNA1073523 (https://www.ebi.ac.uk/ena/browser/view/PRJNA1073523) of BioSample ID SAMN39831887 (https://www.ebi.ac.uk/ena/browser/view/SAMN39831887). The workflows used for bioinformatic analyses are available at https://github.com/ngs-fzb/MTBseq_source and https://github.com/ngs-fzb/HaemoSeq. The tool used for haplotype network analyses is available at https://github.com/ricardoStolze/PegasShiny and workflow and notebooks related to GWAS analyses are available at https://github.com/lbartels0/hinf. Related data is available under https://fairdomhub.org/projects/437.
