# Supplementary material for: Revisiting mutational resistance to ampicillin and cefotaxime in Haemophilus influenzae
Source: Genome Med. 2024 Dec 4;16:140. doi: 10.1186/s13073-024-01406-4 (PMC11616347; doi:10.1186/s13073-024-01406-4)
Supplement: Supplementary file 1 — Additional file 1. Supplementary figures. This file contains all Supplementary Figures and the figure captions. Fig. S1 Overview of the cohorts, Fig. S2 Workflow literature review, Fig. S3 Workflow global public cohort, Fig. S4 Workflow GWAS of clinical cohort, Fig. S5 MIC distributions broth microdilution and CLSI clinical breakpoints, Fig. S6 MIC distributions broth microdilution and EUCAST clinical breakpoints, Fig. S7 Ampicillin MICs of group II isolates stratified to methods and cohorts, Fig. S8 Ampicillin MICs of group II sub-groups stratified to different methods, Fig. S9 Circos plots showing the association between PBP3 substitutions and ampicillin/cefotaxime MICs, Fig. S10 Density plot showing the distribution of ftsI mutations, Fig. S11 Distribution of minimum inhibitory concentration based on gradient diffusion strips for the clinical cohort, Fig. S12 Heatmap visualizing the linkage disequilibrium between all amino acid changing variants within the ftsI gene, Fig. S13 Phylogeny of 298 clinical beta-lactamase negative H. influenzae isolates from three European centers (Lübeck, Würzburg and Lisbon), Fig. S14 The haplotype network displaying the 83 combinations of all 44 variants observed in gene ftsI in at least 10 isolates. [file 13073_2024_1406_MOESM1_ESM.zip › Fig S1_study_overview.pdf]

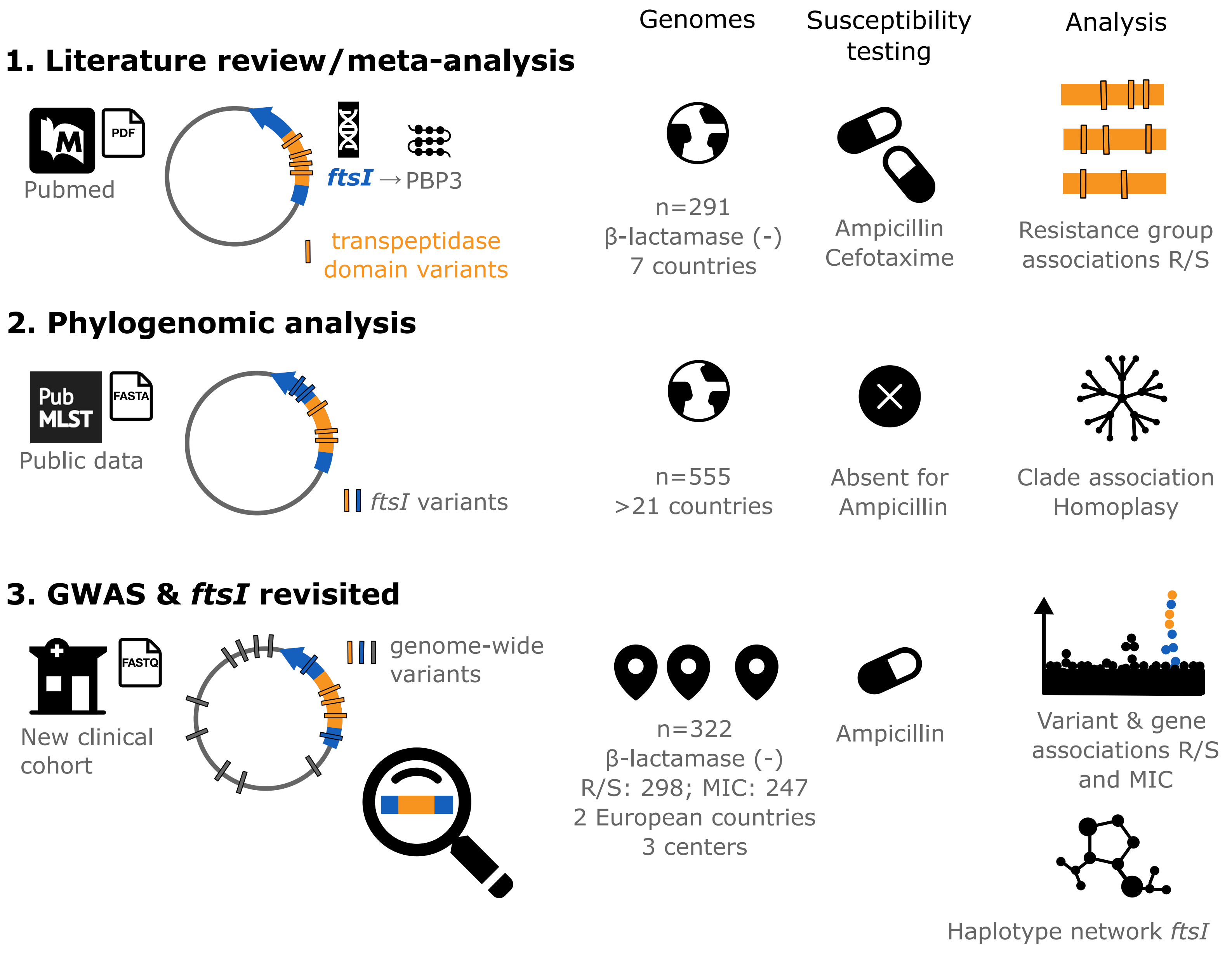
Genomes
Susceptibility testing
Analysis
1. Literature review/meta-analysis
PDF
Pubmed
ftsI → PBP3
transpeptidase domain variants
n=291
β-lactamase (-)
7 countries
Ampicillin
Cefotaxime
Resistance group associations R/S
2. Phylogenomic analysis
Pub MLST
FASTA
Public data
ftsI variants
n=555
>21 countries
Absent for Ampicillin
Clade association
Homoplasy
3. GWAS & ftsI revisited
FASTQ
New clinical cohort
genome-wide variants
n=322
β-lactamase (-)
R/S: 298; MIC: 247
2 European countries
3 centers
Ampicillin
Variant & gene associations R/S and MIC
Haplotype network ftsI
