# Supplementary material for: Revisiting mutational resistance to ampicillin and cefotaxime in Haemophilus influenzae
Source: Genome Med. 2024 Dec 4;16:140. doi: 10.1186/s13073-024-01406-4 (PMC11616347; doi:10.1186/s13073-024-01406-4)
Supplement: Supplementary file 1 — Additional file 1. Supplementary figures. This file contains all Supplementary Figures and the figure captions. Fig. S1 Overview of the cohorts, Fig. S2 Workflow literature review, Fig. S3 Workflow global public cohort, Fig. S4 Workflow GWAS of clinical cohort, Fig. S5 MIC distributions broth microdilution and CLSI clinical breakpoints, Fig. S6 MIC distributions broth microdilution and EUCAST clinical breakpoints, Fig. S7 Ampicillin MICs of group II isolates stratified to methods and cohorts, Fig. S8 Ampicillin MICs of group II sub-groups stratified to different methods, Fig. S9 Circos plots showing the association between PBP3 substitutions and ampicillin/cefotaxime MICs, Fig. S10 Density plot showing the distribution of ftsI mutations, Fig. S11 Distribution of minimum inhibitory concentration based on gradient diffusion strips for the clinical cohort, Fig. S12 Heatmap visualizing the linkage disequilibrium between all amino acid changing variants within the ftsI gene, Fig. S13 Phylogeny of 298 clinical beta-lactamase negative H. influenzae isolates from three European centers (Lübeck, Würzburg and Lisbon), Fig. S14 The haplotype network displaying the 83 combinations of all 44 variants observed in gene ftsI in at least 10 isolates. [file 13073_2024_1406_MOESM1_ESM.zip › Fig S13_Figure_HLRtree298.pdf]

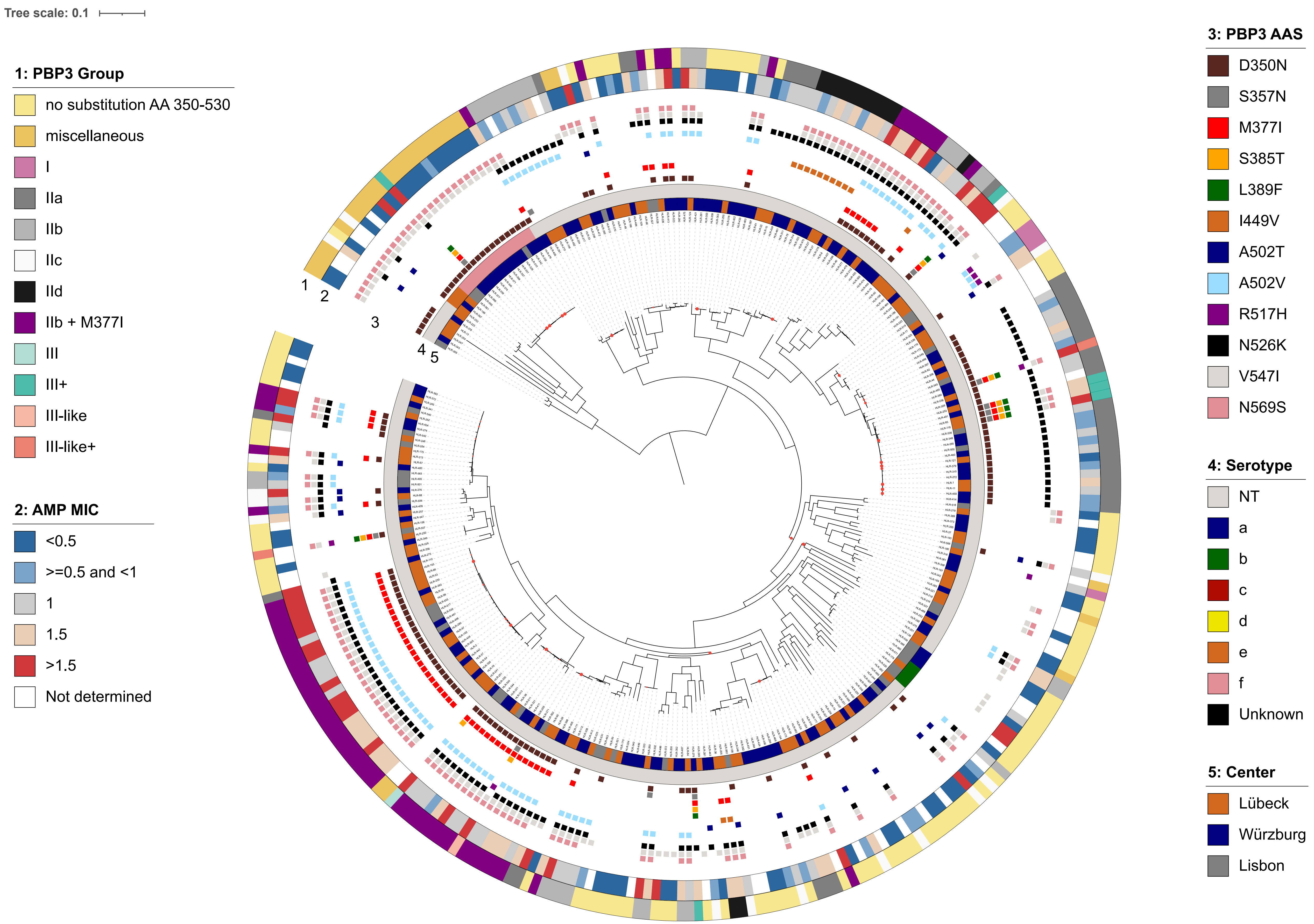

Tree scale: 0.1
1: PBP3 Group
no substitution AA 350-530
miscellaneous
I
IIa
IIb
IIc
IId
IIb + M377I
III
III+
III-like
III-like+
2: AMP MIC
<0.5
>=0.5 and <1
1
1.5
>1.5
Not determined
3: PBP3 AAS
D350N
S357N
M377I
S385T
L389F
I449V
A502T
A502V
R517H
N526K
V547I
N569S
4: Serotype
NT
a
b
c
d
e
f
Unknown
5: Center
Lübeck
Würzburg
Lisbon
1
2
3
4
5
