# Supplementary figures and images for: Revisiting mutational resistance to ampicillin and cefotaxime in Haemophilus influenzae
Source: Genome Med. 2024 Dec 4;16:140. doi: 10.1186/s13073-024-01406-4 (PMC11616347; doi:10.1186/s13073-024-01406-4)

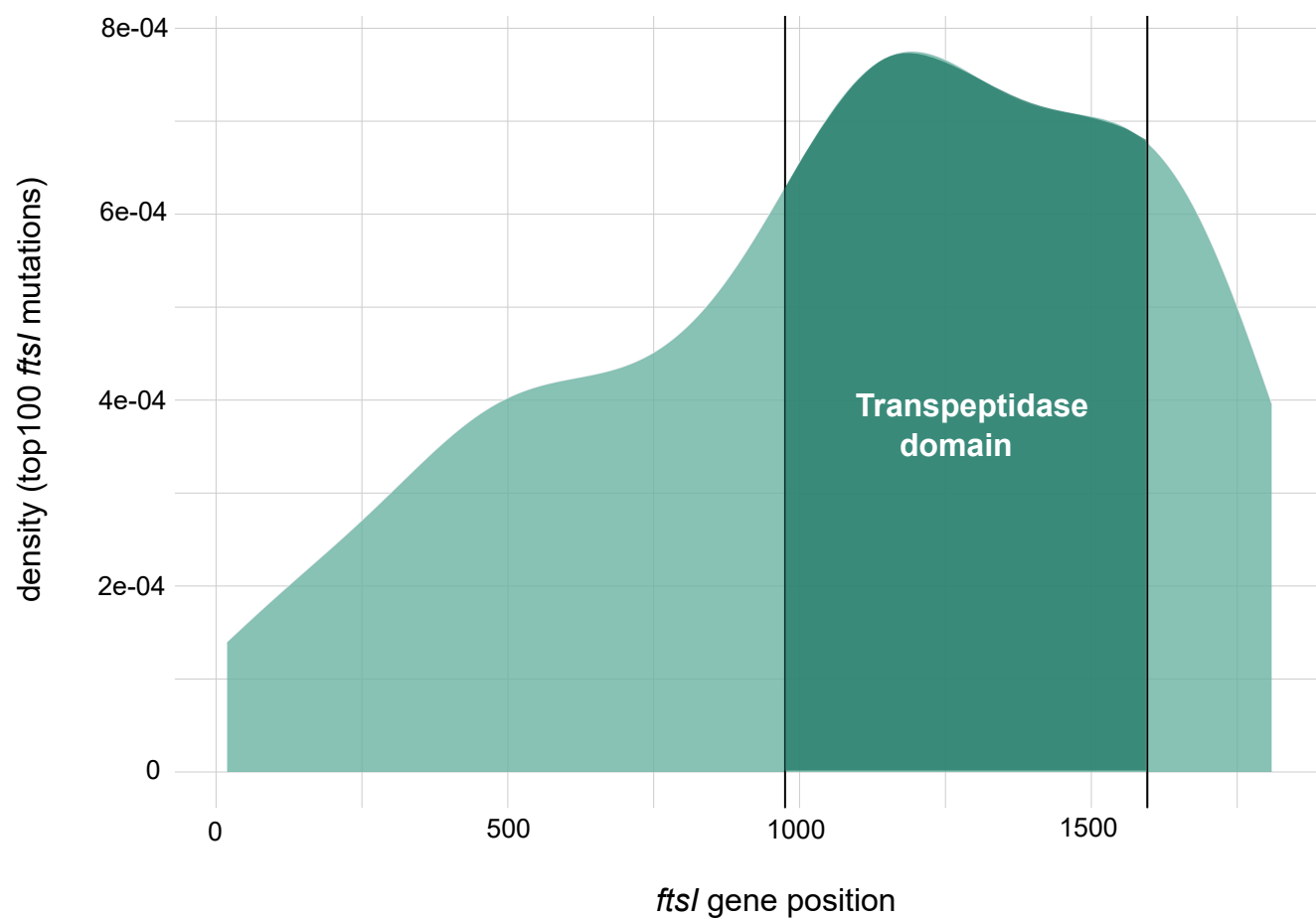

Supplement: Supplementary file 1 — Additional file 1. Supplementary figures. This file contains all Supplementary Figures and the figure captions. Fig. S1 Overview of the cohorts, Fig. S2 Workflow literature review, Fig. S3 Workflow global public cohort, Fig. S4 Workflow GWAS of clinical cohort, Fig. S5 MIC distributions broth microdilution and CLSI clinical breakpoints, Fig. S6 MIC distributions broth microdilution and EUCAST clinical breakpoints, Fig. S7 Ampicillin MICs of group II isolates stratified to methods and cohorts, Fig. S8 Ampicillin MICs of group II sub-groups stratified to different methods, Fig. S9 Circos plots showing the association between PBP3 substitutions and ampicillin/cefotaxime MICs, Fig. S10 Density plot showing the distribution of ftsI mutations, Fig. S11 Distribution of minimum inhibitory concentration based on gradient diffusion strips for the clinical cohort, Fig. S12 Heatmap visualizing the linkage disequilibrium between all amino acid changing variants within the ftsI gene, Fig. S13 Phylogeny of 298 clinical beta-lactamase negative H. influenzae isolates from three European centers (Lübeck, Würzburg and Lisbon), Fig. S14 The haplotype network displaying the 83 combinations of all 44 variants observed in gene ftsI in at least 10 isolates. [file 13073_2024_1406_MOESM1_ESM.zip › Fig S10_Suppl_Figure_ftsI_density_top100_index_2023-12-14.pdf]

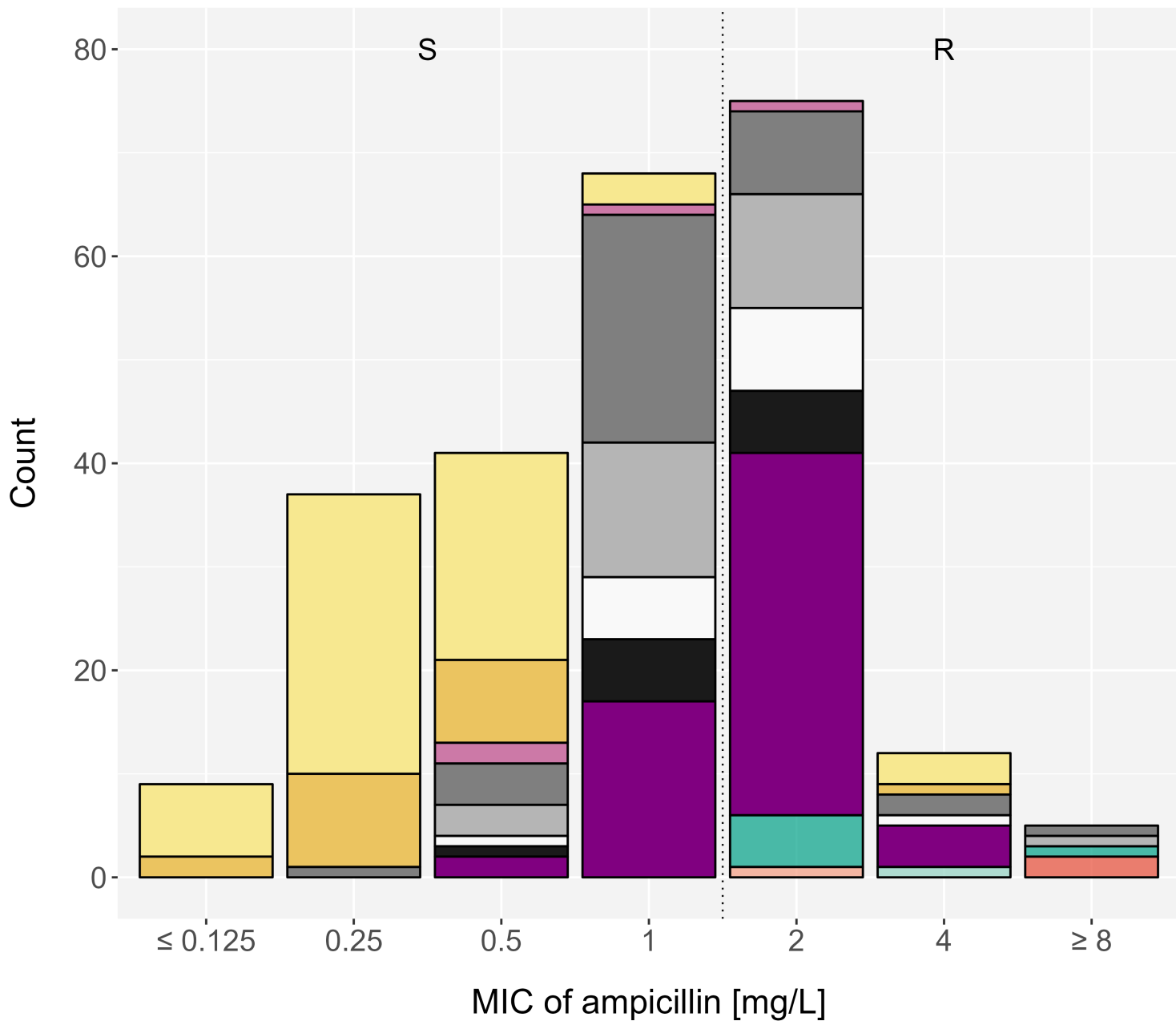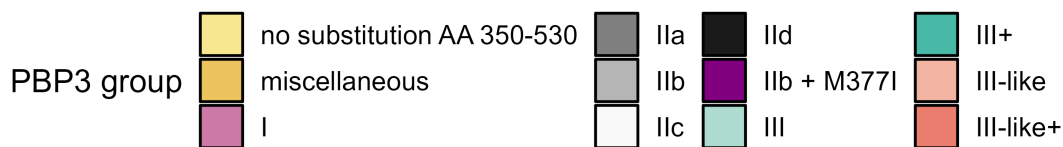

Supplement: Supplementary file 1 — Additional file 1. Supplementary figures. This file contains all Supplementary Figures and the figure captions. Fig. S1 Overview of the cohorts, Fig. S2 Workflow literature review, Fig. S3 Workflow global public cohort, Fig. S4 Workflow GWAS of clinical cohort, Fig. S5 MIC distributions broth microdilution and CLSI clinical breakpoints, Fig. S6 MIC distributions broth microdilution and EUCAST clinical breakpoints, Fig. S7 Ampicillin MICs of group II isolates stratified to methods and cohorts, Fig. S8 Ampicillin MICs of group II sub-groups stratified to different methods, Fig. S9 Circos plots showing the association between PBP3 substitutions and ampicillin/cefotaxime MICs, Fig. S10 Density plot showing the distribution of ftsI mutations, Fig. S11 Distribution of minimum inhibitory concentration based on gradient diffusion strips for the clinical cohort, Fig. S12 Heatmap visualizing the linkage disequilibrium between all amino acid changing variants within the ftsI gene, Fig. S13 Phylogeny of 298 clinical beta-lactamase negative H. influenzae isolates from three European centers (Lübeck, Würzburg and Lisbon), Fig. S14 The haplotype network displaying the 83 combinations of all 44 variants observed in gene ftsI in at least 10 isolates. [file 13073_2024_1406_MOESM1_ESM.zip › Fig S11_Suppl_Figure_MIC_distribution_HLR.pdf]

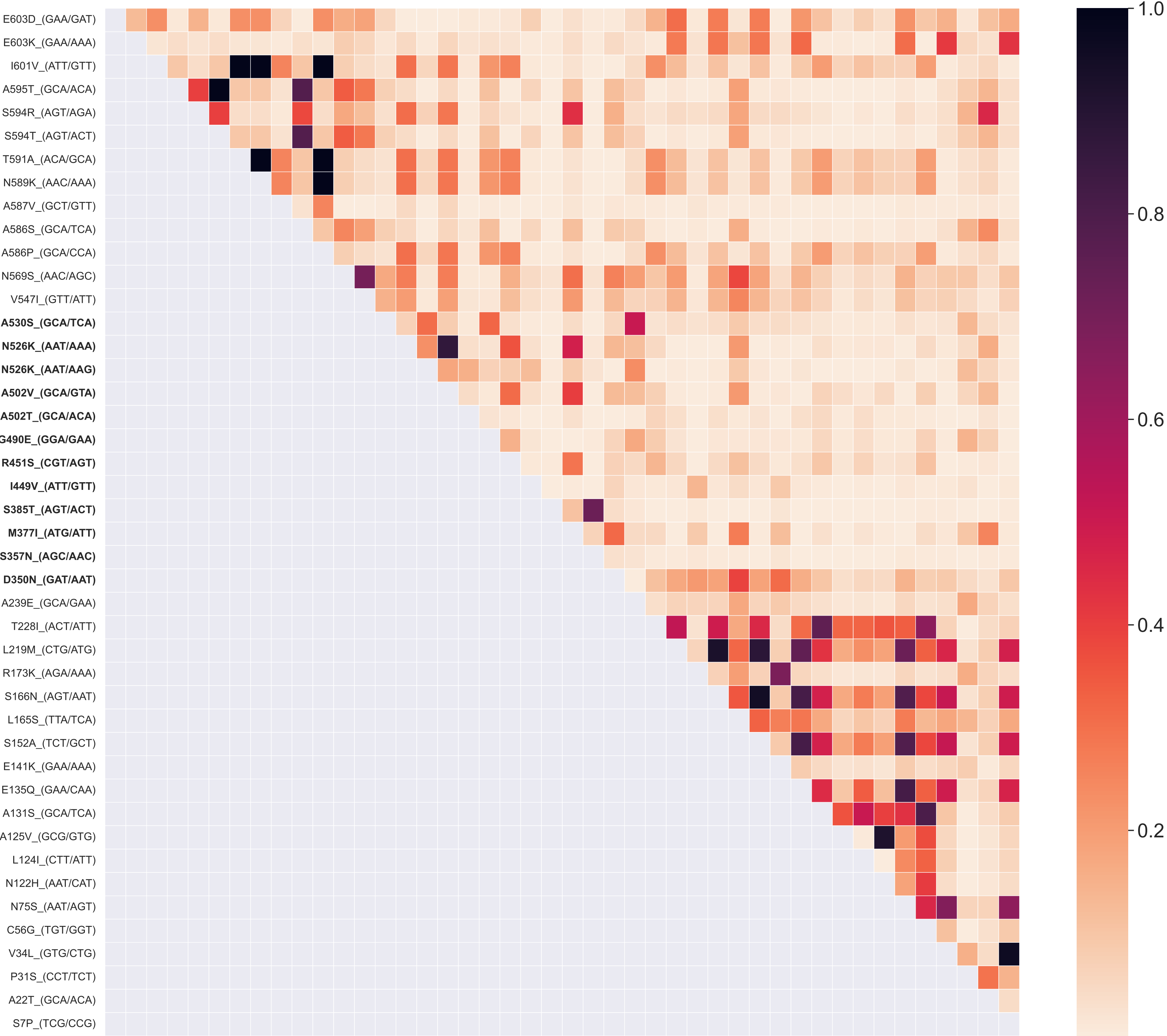

Supplement: Supplementary file 1 — Additional file 1. Supplementary figures. This file contains all Supplementary Figures and the figure captions. Fig. S1 Overview of the cohorts, Fig. S2 Workflow literature review, Fig. S3 Workflow global public cohort, Fig. S4 Workflow GWAS of clinical cohort, Fig. S5 MIC distributions broth microdilution and CLSI clinical breakpoints, Fig. S6 MIC distributions broth microdilution and EUCAST clinical breakpoints, Fig. S7 Ampicillin MICs of group II isolates stratified to methods and cohorts, Fig. S8 Ampicillin MICs of group II sub-groups stratified to different methods, Fig. S9 Circos plots showing the association between PBP3 substitutions and ampicillin/cefotaxime MICs, Fig. S10 Density plot showing the distribution of ftsI mutations, Fig. S11 Distribution of minimum inhibitory concentration based on gradient diffusion strips for the clinical cohort, Fig. S12 Heatmap visualizing the linkage disequilibrium between all amino acid changing variants within the ftsI gene, Fig. S13 Phylogeny of 298 clinical beta-lactamase negative H. influenzae isolates from three European centers (Lübeck, Würzburg and Lisbon), Fig. S14 The haplotype network displaying the 83 combinations of all 44 variants observed in gene ftsI in at least 10 isolates. [file 13073_2024_1406_MOESM1_ESM.zip › Fig S12_Suppl_Figure_ld_ftsI.pdf]

**A**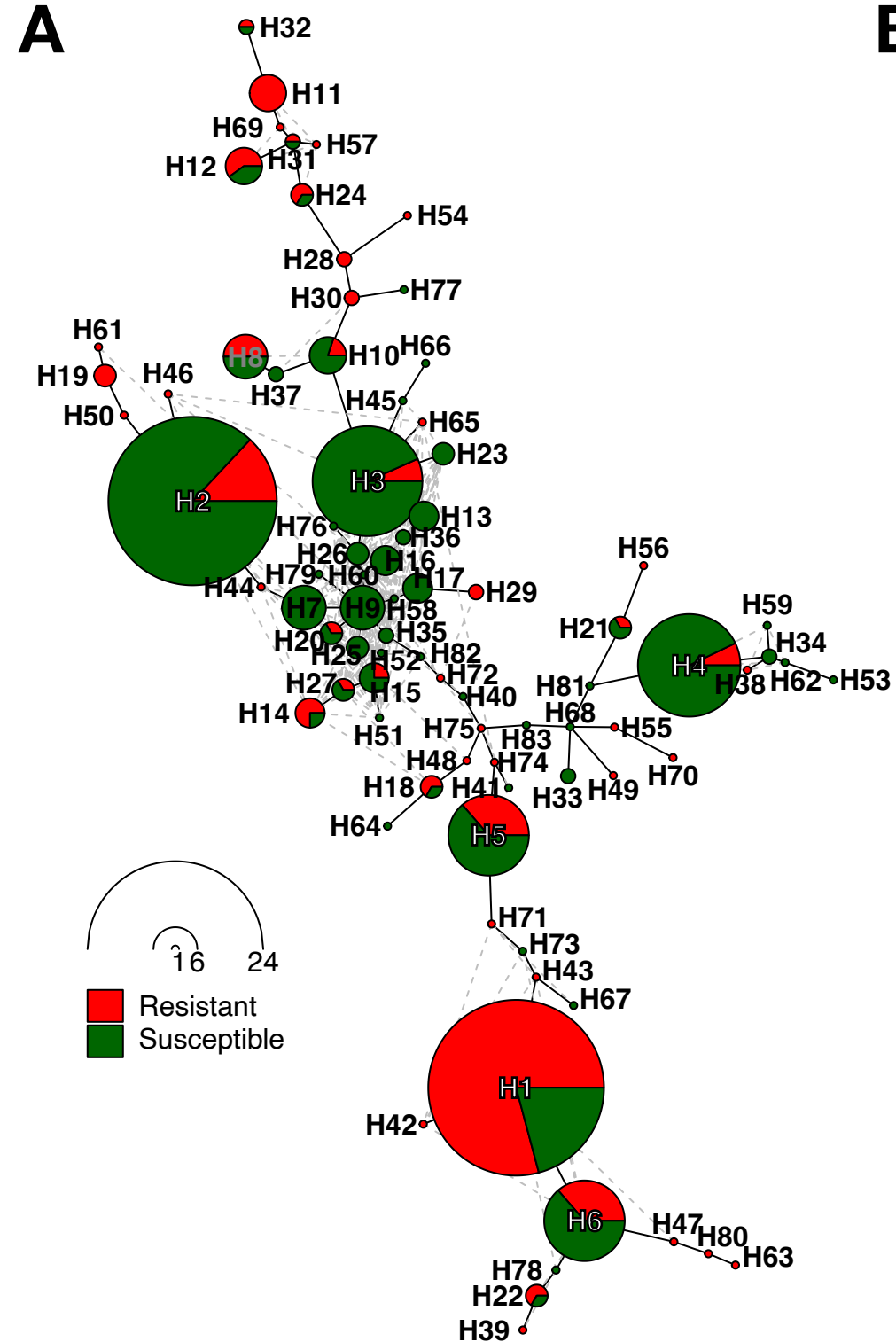**B**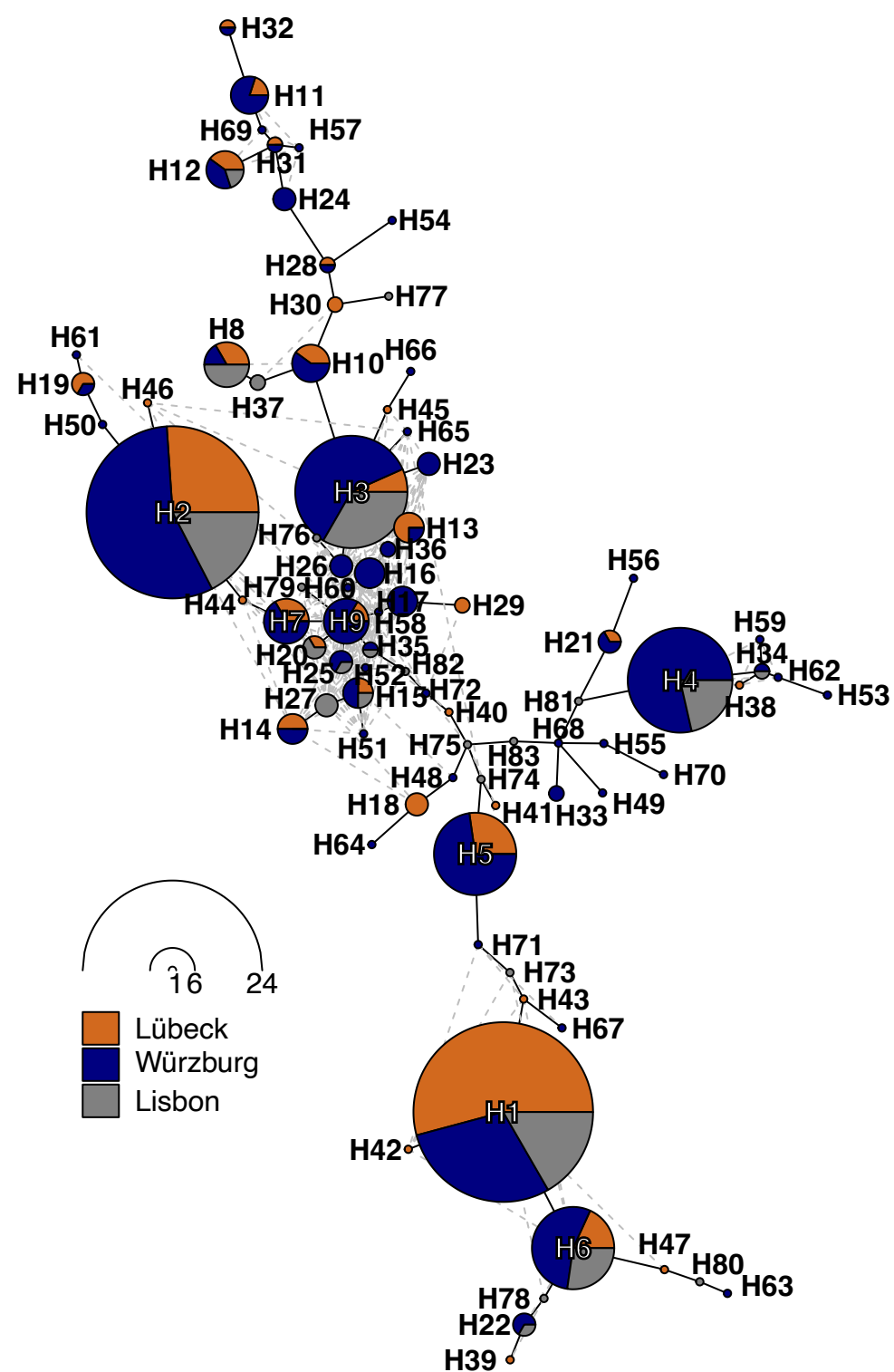**C**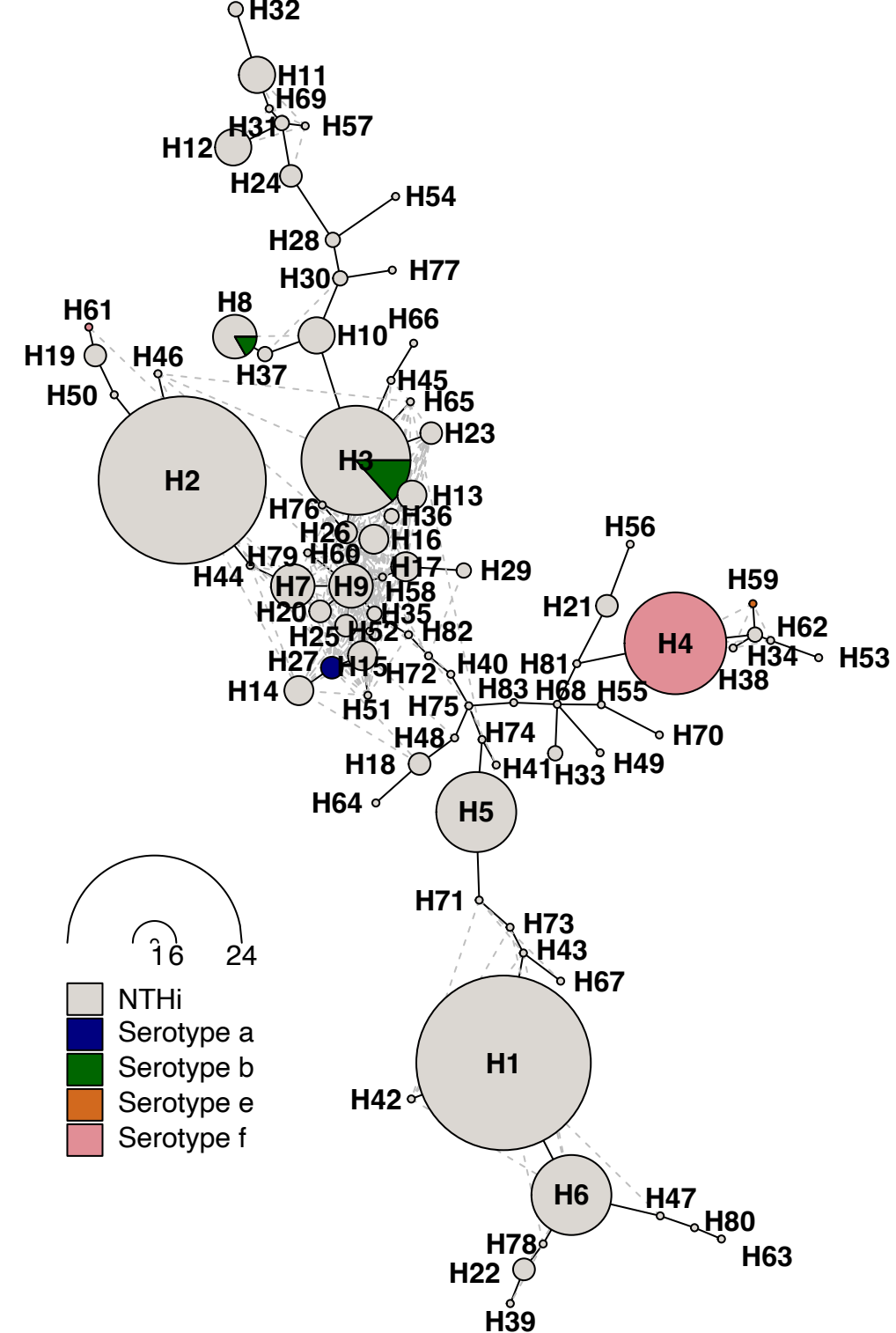

Supplement: Supplementary file 1 — Additional file 1. Supplementary figures. This file contains all Supplementary Figures and the figure captions. Fig. S1 Overview of the cohorts, Fig. S2 Workflow literature review, Fig. S3 Workflow global public cohort, Fig. S4 Workflow GWAS of clinical cohort, Fig. S5 MIC distributions broth microdilution and CLSI clinical breakpoints, Fig. S6 MIC distributions broth microdilution and EUCAST clinical breakpoints, Fig. S7 Ampicillin MICs of group II isolates stratified to methods and cohorts, Fig. S8 Ampicillin MICs of group II sub-groups stratified to different methods, Fig. S9 Circos plots showing the association between PBP3 substitutions and ampicillin/cefotaxime MICs, Fig. S10 Density plot showing the distribution of ftsI mutations, Fig. S11 Distribution of minimum inhibitory concentration based on gradient diffusion strips for the clinical cohort, Fig. S12 Heatmap visualizing the linkage disequilibrium between all amino acid changing variants within the ftsI gene, Fig. S13 Phylogeny of 298 clinical beta-lactamase negative H. influenzae isolates from three European centers (Lübeck, Würzburg and Lisbon), Fig. S14 The haplotype network displaying the 83 combinations of all 44 variants observed in gene ftsI in at least 10 isolates. [file 13073_2024_1406_MOESM1_ESM.zip › Fig S14_Suppl_Figure_network.pdf]

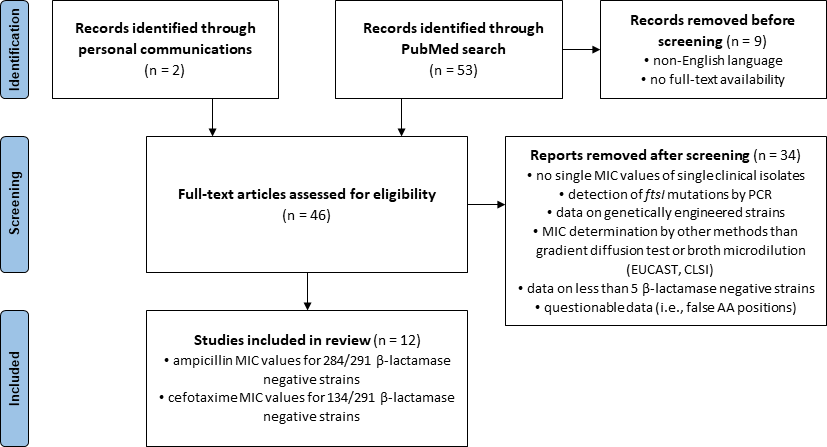

Supplement: Supplementary file 1 — Additional file 1. Supplementary figures. This file contains all Supplementary Figures and the figure captions. Fig. S1 Overview of the cohorts, Fig. S2 Workflow literature review, Fig. S3 Workflow global public cohort, Fig. S4 Workflow GWAS of clinical cohort, Fig. S5 MIC distributions broth microdilution and CLSI clinical breakpoints, Fig. S6 MIC distributions broth microdilution and EUCAST clinical breakpoints, Fig. S7 Ampicillin MICs of group II isolates stratified to methods and cohorts, Fig. S8 Ampicillin MICs of group II sub-groups stratified to different methods, Fig. S9 Circos plots showing the association between PBP3 substitutions and ampicillin/cefotaxime MICs, Fig. S10 Density plot showing the distribution of ftsI mutations, Fig. S11 Distribution of minimum inhibitory concentration based on gradient diffusion strips for the clinical cohort, Fig. S12 Heatmap visualizing the linkage disequilibrium between all amino acid changing variants within the ftsI gene, Fig. S13 Phylogeny of 298 clinical beta-lactamase negative H. influenzae isolates from three European centers (Lübeck, Würzburg and Lisbon), Fig. S14 The haplotype network displaying the 83 combinations of all 44 variants observed in gene ftsI in at least 10 isolates. [file 13073_2024_1406_MOESM1_ESM.zip › Fig S2_Suppl_Figure_literature_review_workflow_diagram.png]

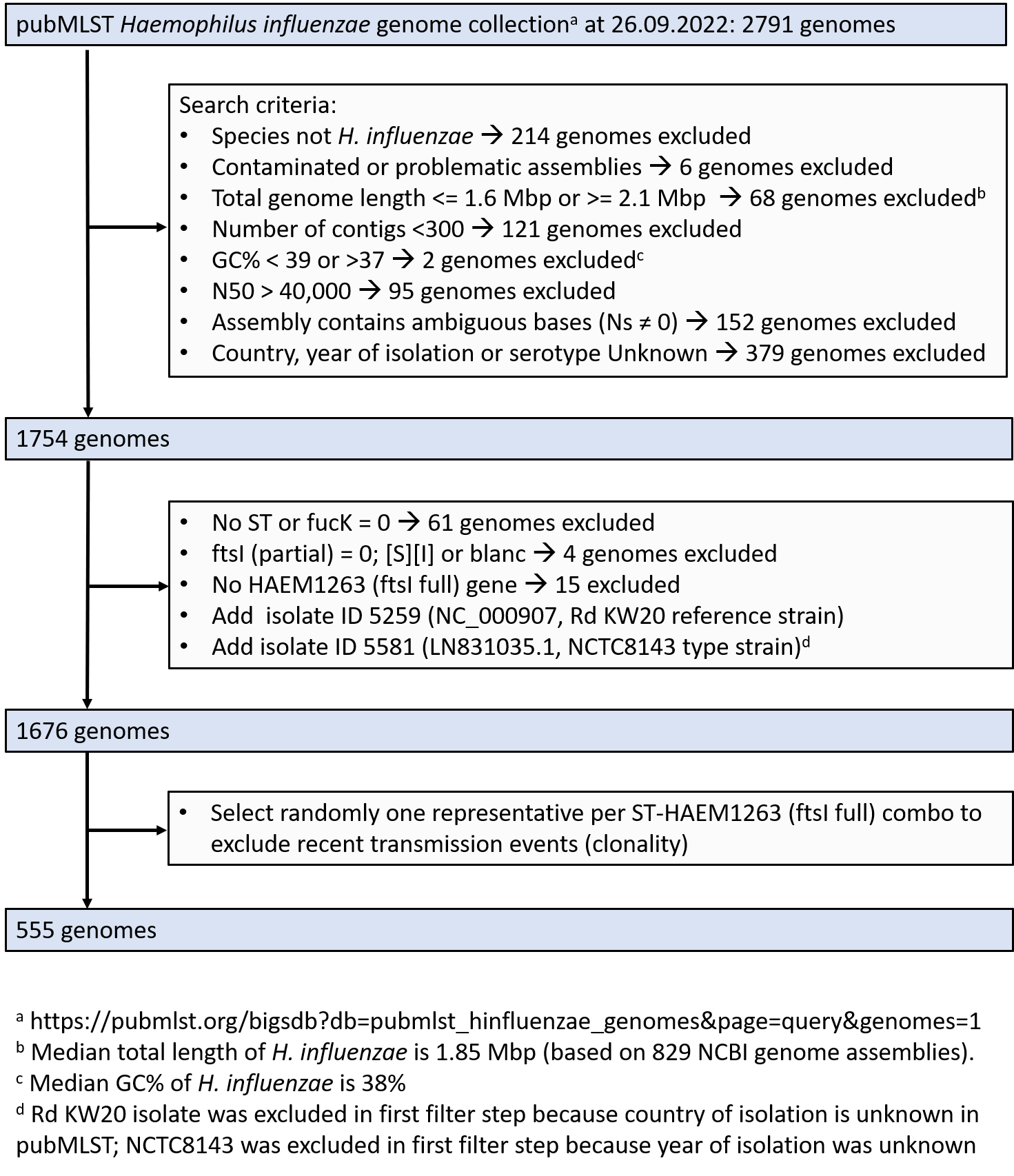

Supplement: Supplementary file 1 — Additional file 1. Supplementary figures. This file contains all Supplementary Figures and the figure captions. Fig. S1 Overview of the cohorts, Fig. S2 Workflow literature review, Fig. S3 Workflow global public cohort, Fig. S4 Workflow GWAS of clinical cohort, Fig. S5 MIC distributions broth microdilution and CLSI clinical breakpoints, Fig. S6 MIC distributions broth microdilution and EUCAST clinical breakpoints, Fig. S7 Ampicillin MICs of group II isolates stratified to methods and cohorts, Fig. S8 Ampicillin MICs of group II sub-groups stratified to different methods, Fig. S9 Circos plots showing the association between PBP3 substitutions and ampicillin/cefotaxime MICs, Fig. S10 Density plot showing the distribution of ftsI mutations, Fig. S11 Distribution of minimum inhibitory concentration based on gradient diffusion strips for the clinical cohort, Fig. S12 Heatmap visualizing the linkage disequilibrium between all amino acid changing variants within the ftsI gene, Fig. S13 Phylogeny of 298 clinical beta-lactamase negative H. influenzae isolates from three European centers (Lübeck, Würzburg and Lisbon), Fig. S14 The haplotype network displaying the 83 combinations of all 44 variants observed in gene ftsI in at least 10 isolates. [file 13073_2024_1406_MOESM1_ESM.zip › Fig S3_Supplementary_Figure_WorkflowPubMLSTSelection.tif]

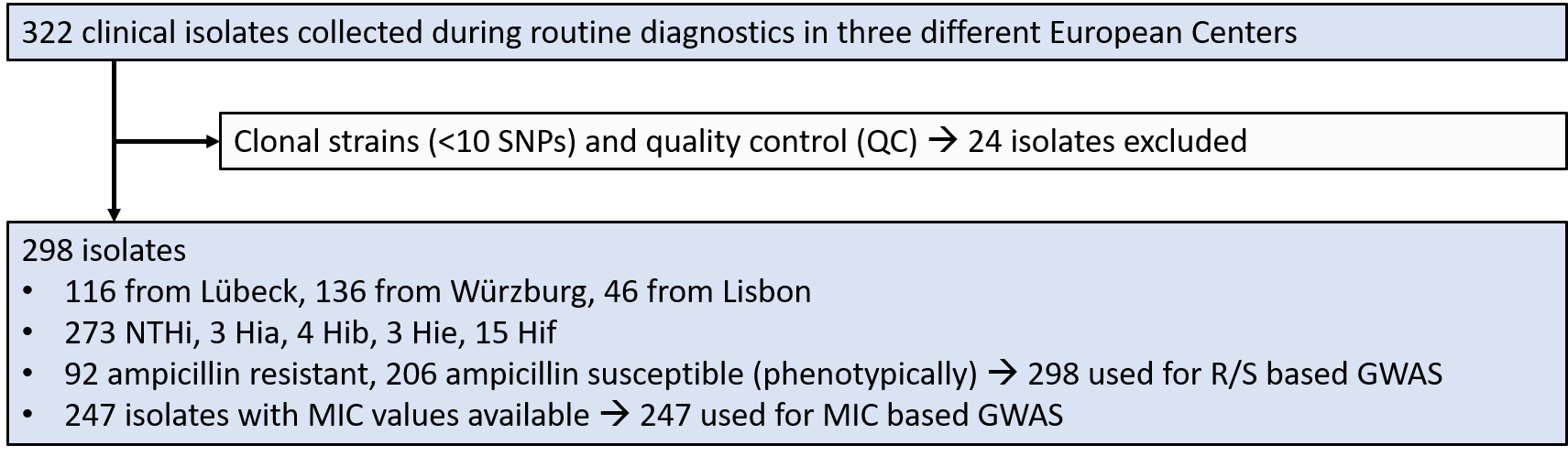

Supplement: Supplementary file 1 — Additional file 1. Supplementary figures. This file contains all Supplementary Figures and the figure captions. Fig. S1 Overview of the cohorts, Fig. S2 Workflow literature review, Fig. S3 Workflow global public cohort, Fig. S4 Workflow GWAS of clinical cohort, Fig. S5 MIC distributions broth microdilution and CLSI clinical breakpoints, Fig. S6 MIC distributions broth microdilution and EUCAST clinical breakpoints, Fig. S7 Ampicillin MICs of group II isolates stratified to methods and cohorts, Fig. S8 Ampicillin MICs of group II sub-groups stratified to different methods, Fig. S9 Circos plots showing the association between PBP3 substitutions and ampicillin/cefotaxime MICs, Fig. S10 Density plot showing the distribution of ftsI mutations, Fig. S11 Distribution of minimum inhibitory concentration based on gradient diffusion strips for the clinical cohort, Fig. S12 Heatmap visualizing the linkage disequilibrium between all amino acid changing variants within the ftsI gene, Fig. S13 Phylogeny of 298 clinical beta-lactamase negative H. influenzae isolates from three European centers (Lübeck, Würzburg and Lisbon), Fig. S14 The haplotype network displaying the 83 combinations of all 44 variants observed in gene ftsI in at least 10 isolates. [file 13073_2024_1406_MOESM1_ESM.zip › Fig S4_Supplementary_Figure_WorkflowClinicalCohorSelection.tif]

**A**

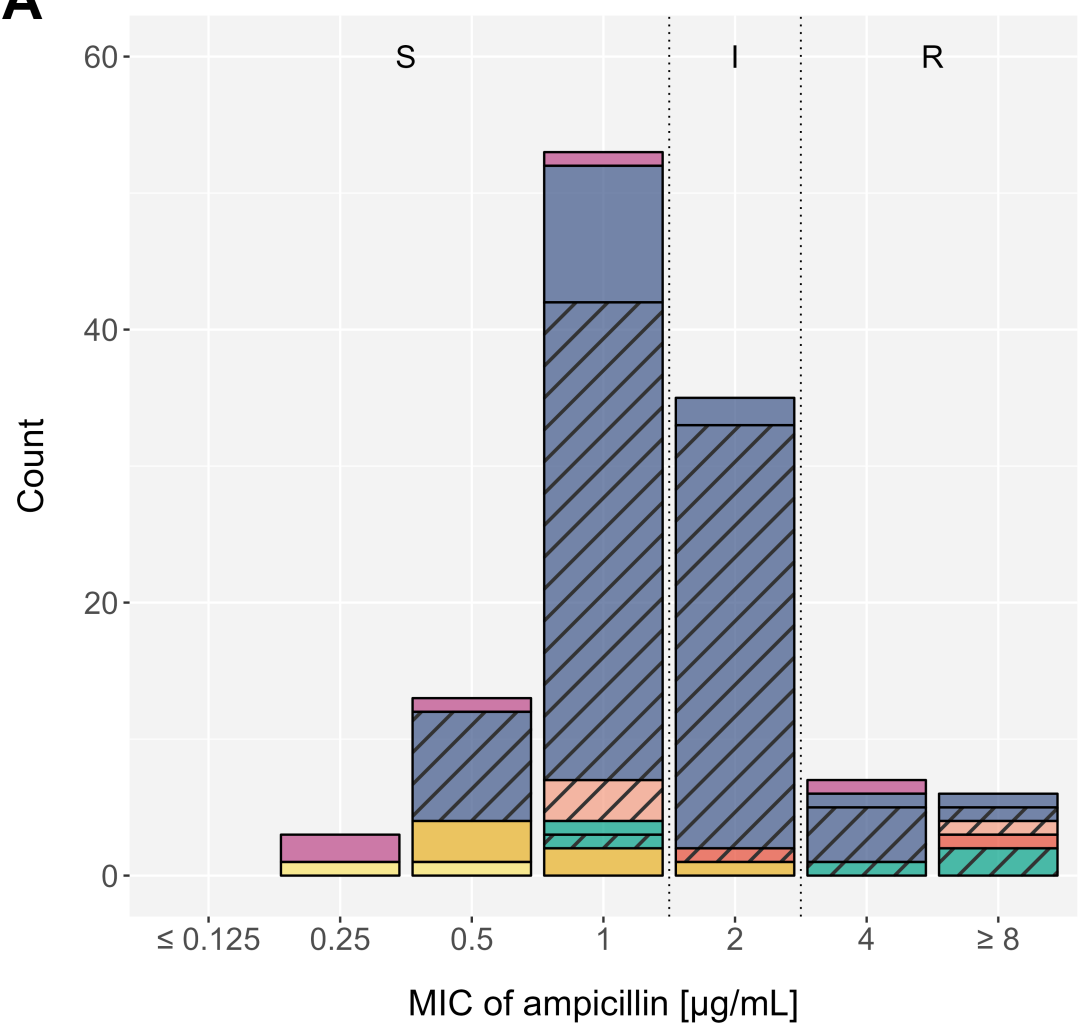

**B**

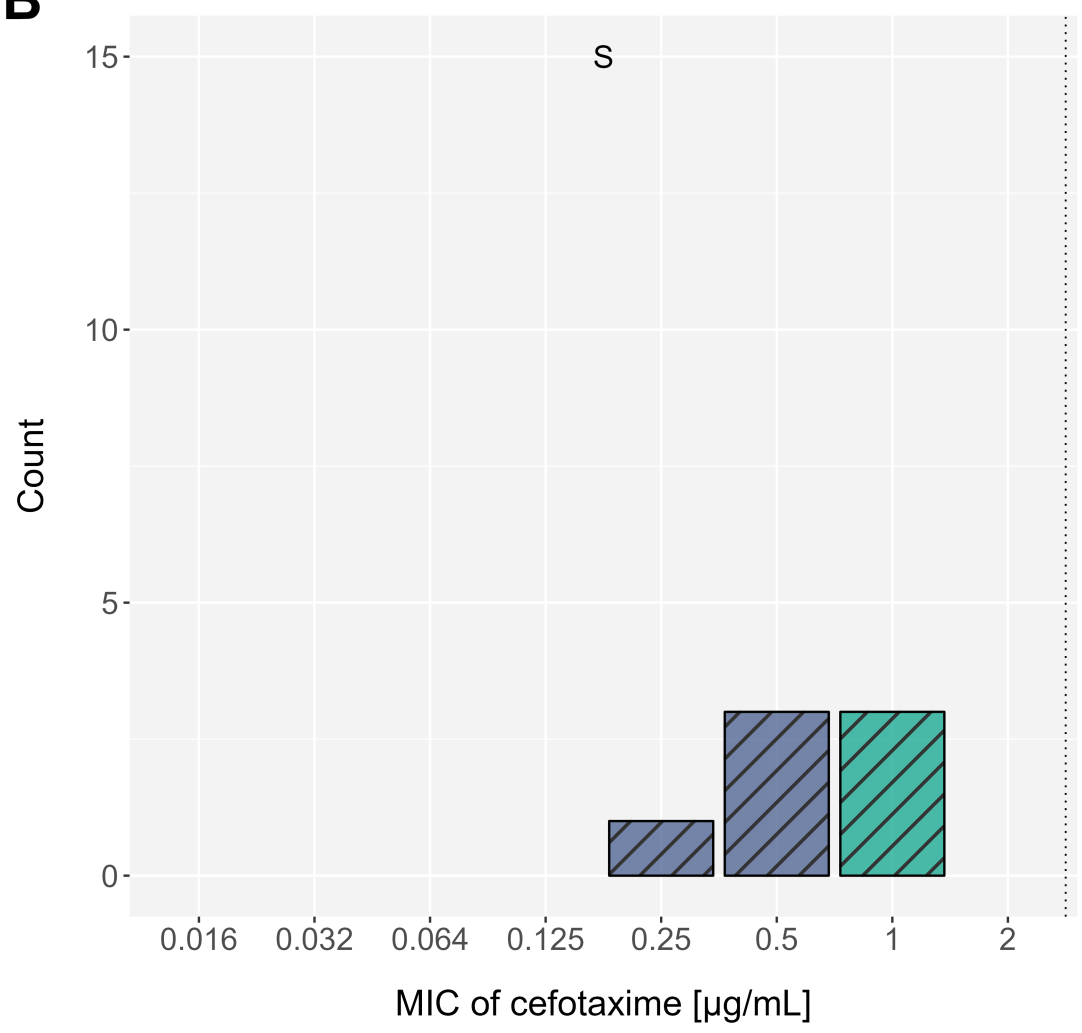

Supplement: Supplementary file 1 — Additional file 1. Supplementary figures. This file contains all Supplementary Figures and the figure captions. Fig. S1 Overview of the cohorts, Fig. S2 Workflow literature review, Fig. S3 Workflow global public cohort, Fig. S4 Workflow GWAS of clinical cohort, Fig. S5 MIC distributions broth microdilution and CLSI clinical breakpoints, Fig. S6 MIC distributions broth microdilution and EUCAST clinical breakpoints, Fig. S7 Ampicillin MICs of group II isolates stratified to methods and cohorts, Fig. S8 Ampicillin MICs of group II sub-groups stratified to different methods, Fig. S9 Circos plots showing the association between PBP3 substitutions and ampicillin/cefotaxime MICs, Fig. S10 Density plot showing the distribution of ftsI mutations, Fig. S11 Distribution of minimum inhibitory concentration based on gradient diffusion strips for the clinical cohort, Fig. S12 Heatmap visualizing the linkage disequilibrium between all amino acid changing variants within the ftsI gene, Fig. S13 Phylogeny of 298 clinical beta-lactamase negative H. influenzae isolates from three European centers (Lübeck, Würzburg and Lisbon), Fig. S14 The haplotype network displaying the 83 combinations of all 44 variants observed in gene ftsI in at least 10 isolates. [file 13073_2024_1406_MOESM1_ESM.zip › Fig S5_Suppl_Figure_MIC_distribution_BM_CLSI.pdf]

**A**

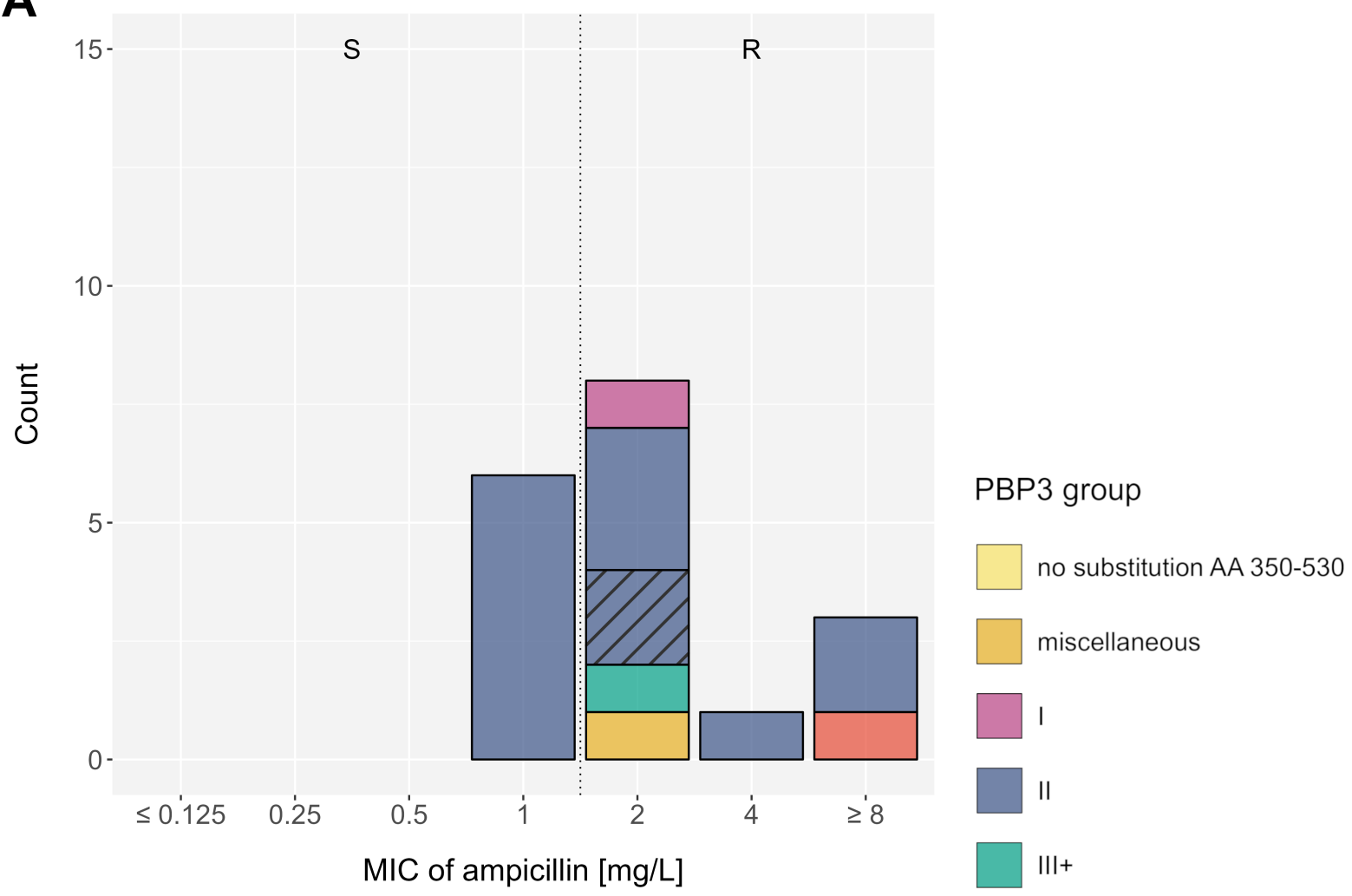

**B**

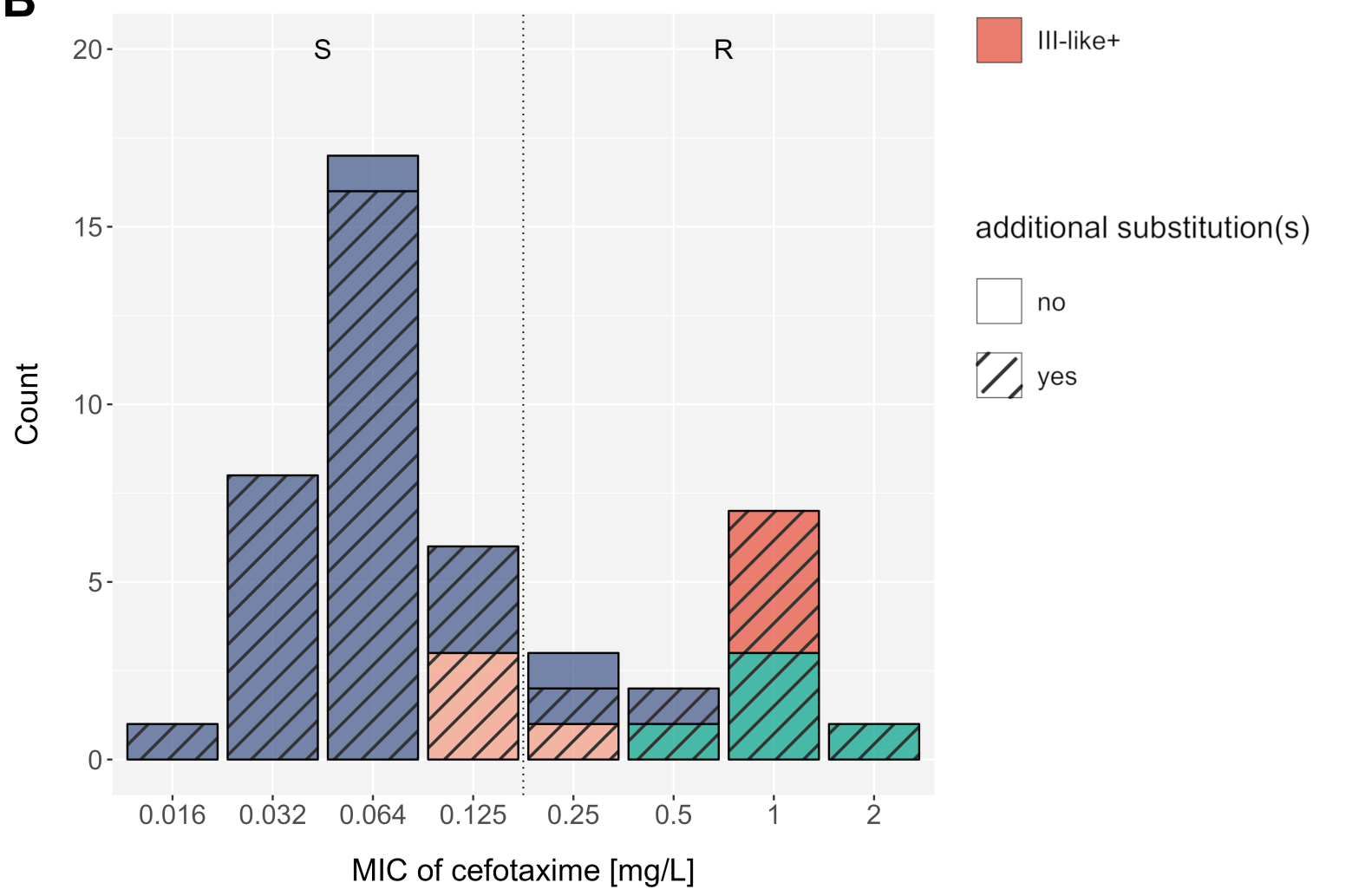

Supplement: Supplementary file 1 — Additional file 1. Supplementary figures. This file contains all Supplementary Figures and the figure captions. Fig. S1 Overview of the cohorts, Fig. S2 Workflow literature review, Fig. S3 Workflow global public cohort, Fig. S4 Workflow GWAS of clinical cohort, Fig. S5 MIC distributions broth microdilution and CLSI clinical breakpoints, Fig. S6 MIC distributions broth microdilution and EUCAST clinical breakpoints, Fig. S7 Ampicillin MICs of group II isolates stratified to methods and cohorts, Fig. S8 Ampicillin MICs of group II sub-groups stratified to different methods, Fig. S9 Circos plots showing the association between PBP3 substitutions and ampicillin/cefotaxime MICs, Fig. S10 Density plot showing the distribution of ftsI mutations, Fig. S11 Distribution of minimum inhibitory concentration based on gradient diffusion strips for the clinical cohort, Fig. S12 Heatmap visualizing the linkage disequilibrium between all amino acid changing variants within the ftsI gene, Fig. S13 Phylogeny of 298 clinical beta-lactamase negative H. influenzae isolates from three European centers (Lübeck, Würzburg and Lisbon), Fig. S14 The haplotype network displaying the 83 combinations of all 44 variants observed in gene ftsI in at least 10 isolates. [file 13073_2024_1406_MOESM1_ESM.zip › Fig S6_Suppl_Figure_MIC_distribution_BM_EUCAST.pdf]

**A**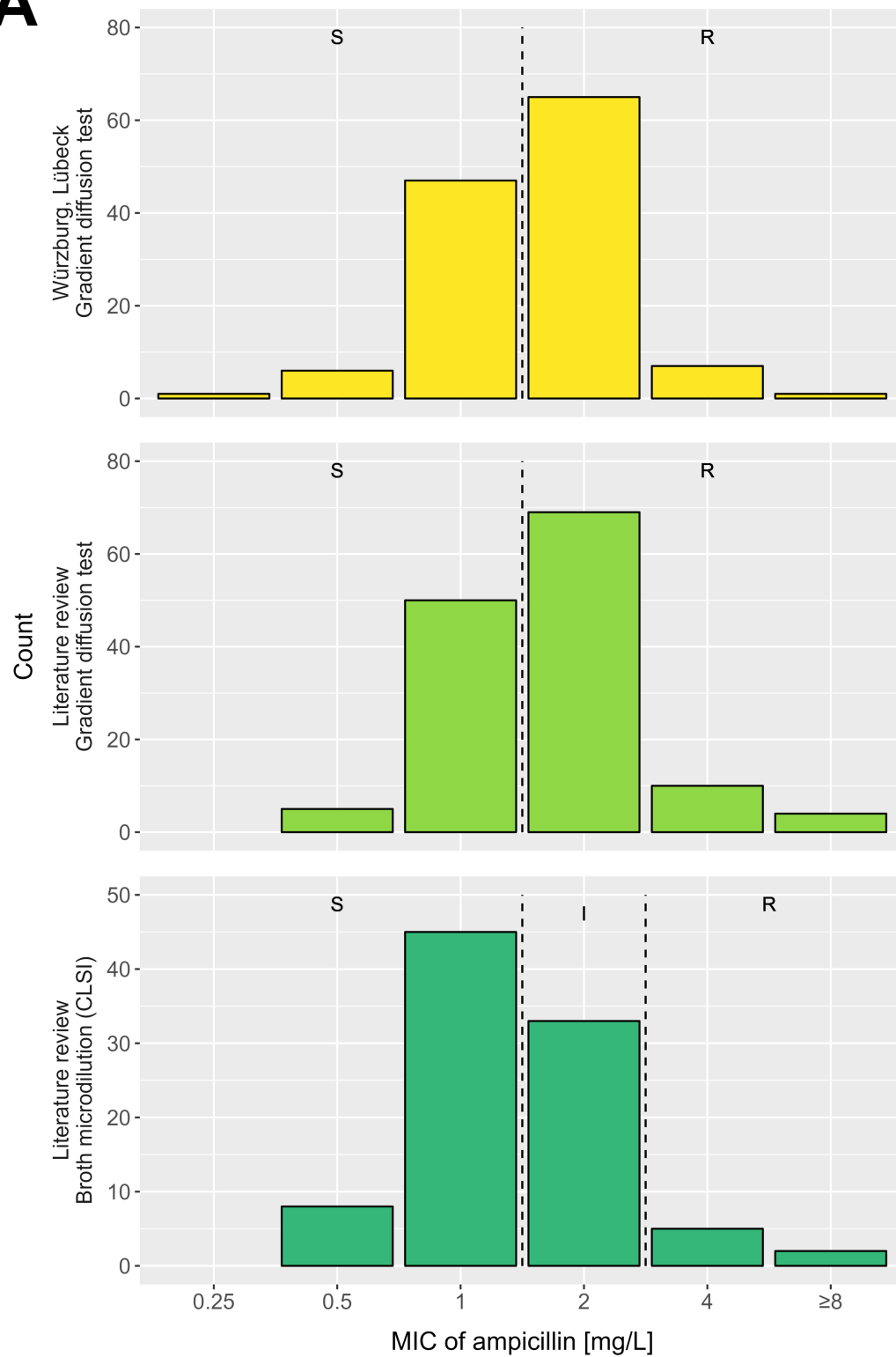**B**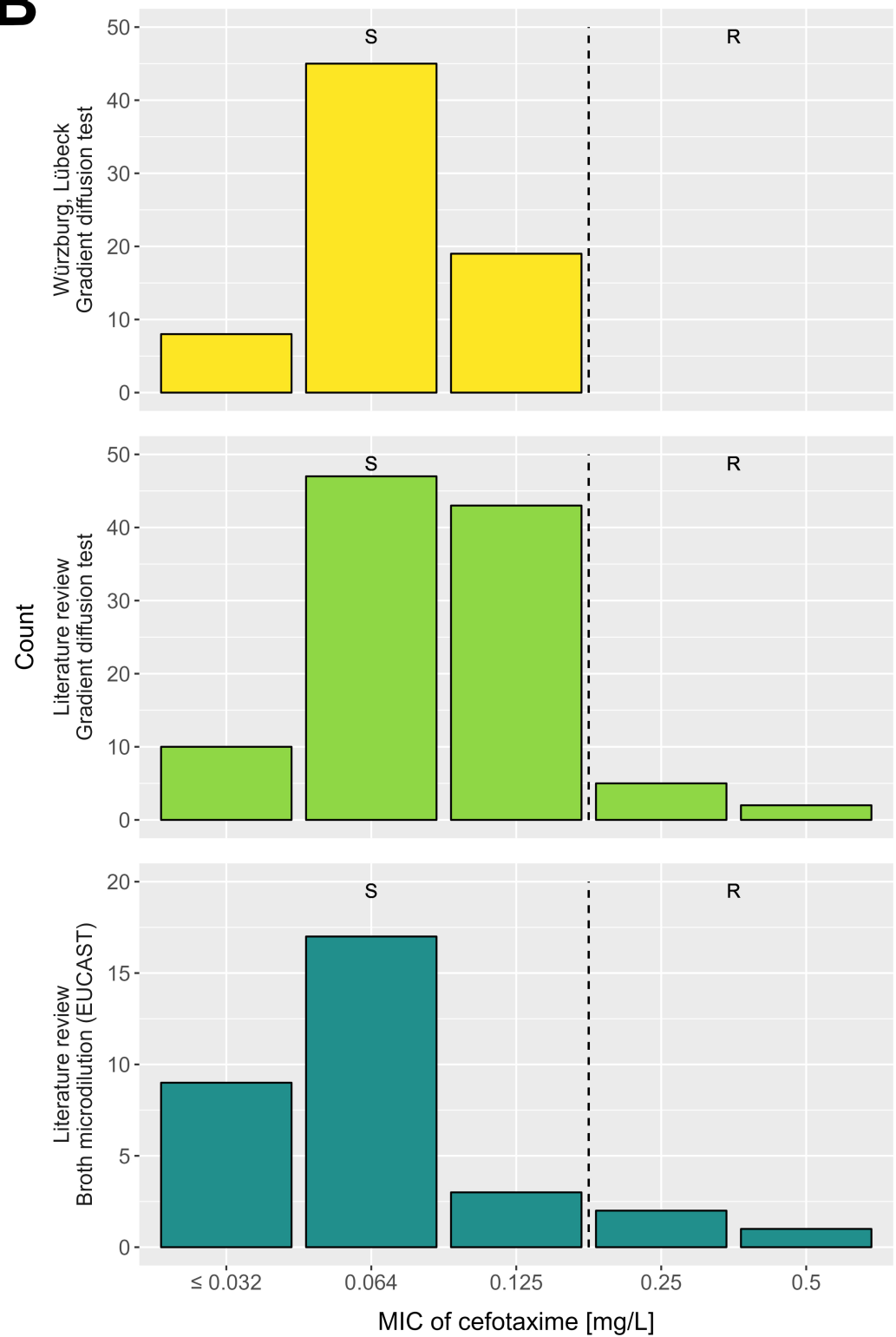

Supplement: Supplementary file 1 — Additional file 1. Supplementary figures. This file contains all Supplementary Figures and the figure captions. Fig. S1 Overview of the cohorts, Fig. S2 Workflow literature review, Fig. S3 Workflow global public cohort, Fig. S4 Workflow GWAS of clinical cohort, Fig. S5 MIC distributions broth microdilution and CLSI clinical breakpoints, Fig. S6 MIC distributions broth microdilution and EUCAST clinical breakpoints, Fig. S7 Ampicillin MICs of group II isolates stratified to methods and cohorts, Fig. S8 Ampicillin MICs of group II sub-groups stratified to different methods, Fig. S9 Circos plots showing the association between PBP3 substitutions and ampicillin/cefotaxime MICs, Fig. S10 Density plot showing the distribution of ftsI mutations, Fig. S11 Distribution of minimum inhibitory concentration based on gradient diffusion strips for the clinical cohort, Fig. S12 Heatmap visualizing the linkage disequilibrium between all amino acid changing variants within the ftsI gene, Fig. S13 Phylogeny of 298 clinical beta-lactamase negative H. influenzae isolates from three European centers (Lübeck, Würzburg and Lisbon), Fig. S14 The haplotype network displaying the 83 combinations of all 44 variants observed in gene ftsI in at least 10 isolates. [file 13073_2024_1406_MOESM1_ESM.zip › Fig S7_Suppl_Figure_Cohort_MIC_distribution_groupII.pdf]

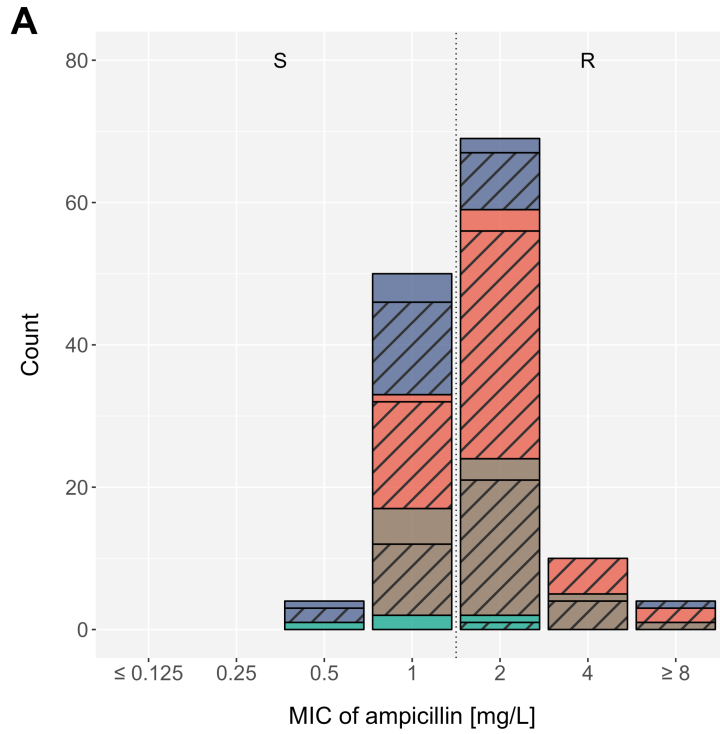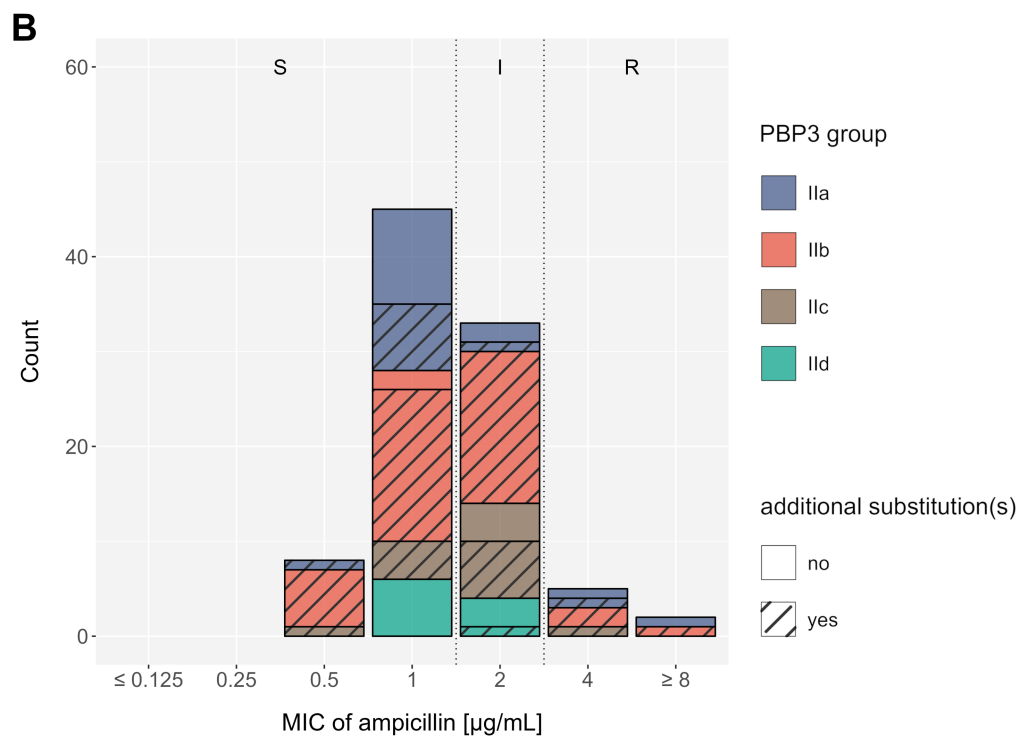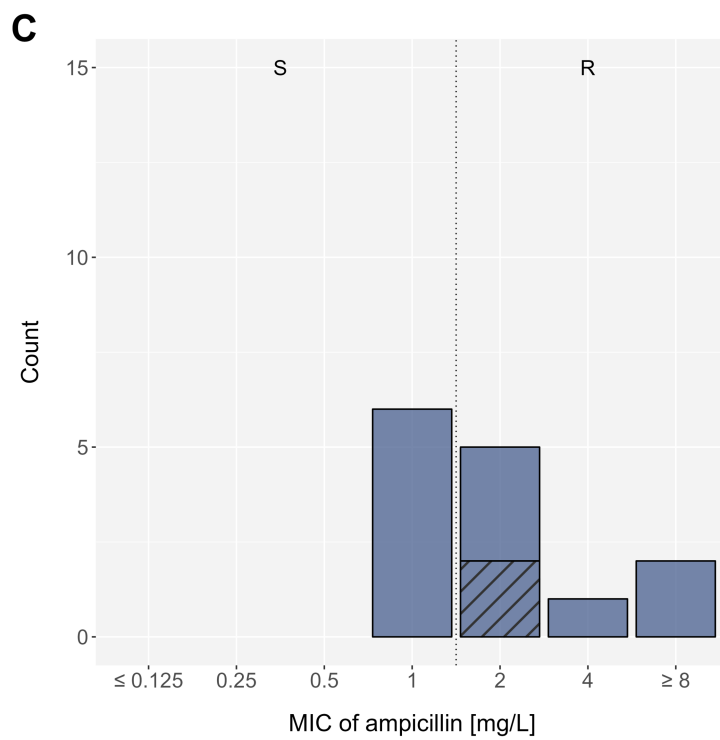

Supplement: Supplementary file 1 — Additional file 1. Supplementary figures. This file contains all Supplementary Figures and the figure captions. Fig. S1 Overview of the cohorts, Fig. S2 Workflow literature review, Fig. S3 Workflow global public cohort, Fig. S4 Workflow GWAS of clinical cohort, Fig. S5 MIC distributions broth microdilution and CLSI clinical breakpoints, Fig. S6 MIC distributions broth microdilution and EUCAST clinical breakpoints, Fig. S7 Ampicillin MICs of group II isolates stratified to methods and cohorts, Fig. S8 Ampicillin MICs of group II sub-groups stratified to different methods, Fig. S9 Circos plots showing the association between PBP3 substitutions and ampicillin/cefotaxime MICs, Fig. S10 Density plot showing the distribution of ftsI mutations, Fig. S11 Distribution of minimum inhibitory concentration based on gradient diffusion strips for the clinical cohort, Fig. S12 Heatmap visualizing the linkage disequilibrium between all amino acid changing variants within the ftsI gene, Fig. S13 Phylogeny of 298 clinical beta-lactamase negative H. influenzae isolates from three European centers (Lübeck, Würzburg and Lisbon), Fig. S14 The haplotype network displaying the 83 combinations of all 44 variants observed in gene ftsI in at least 10 isolates. [file 13073_2024_1406_MOESM1_ESM.zip › Fig S8_Suppl_Figure_MIC_distribution_groupII_plots.pdf]

**A**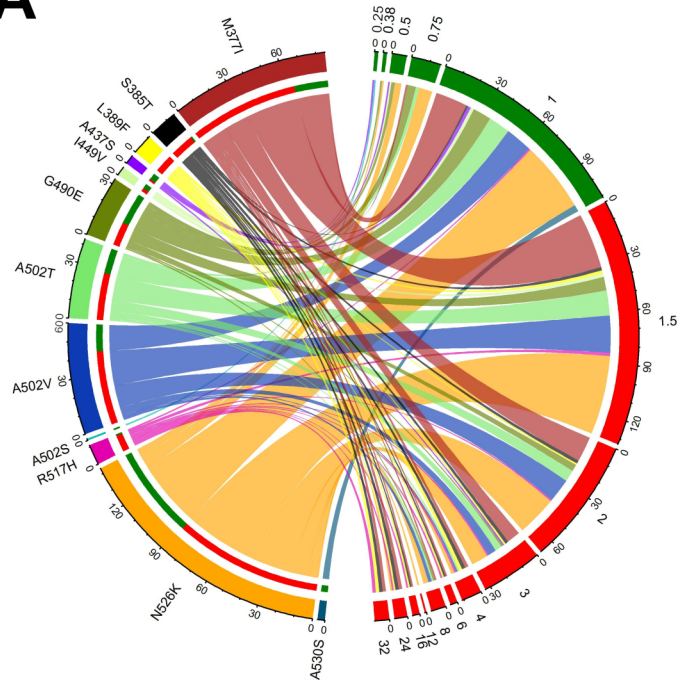**B**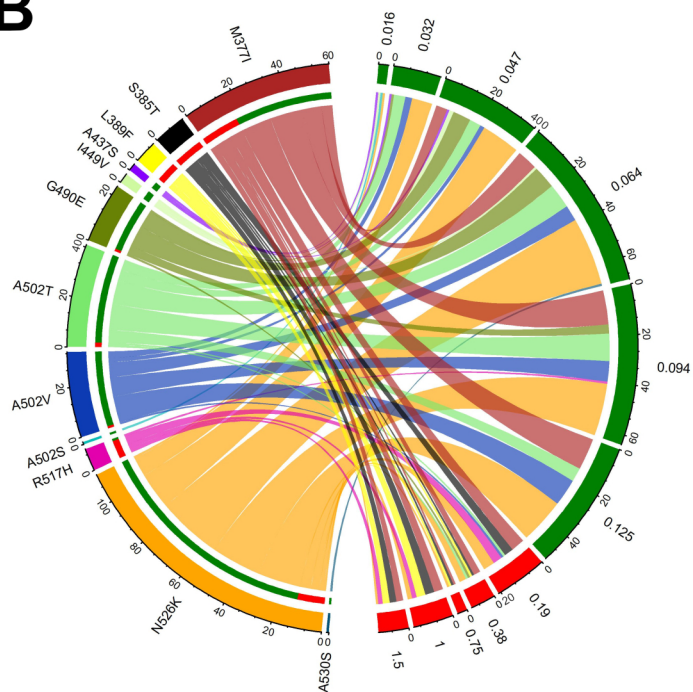**C**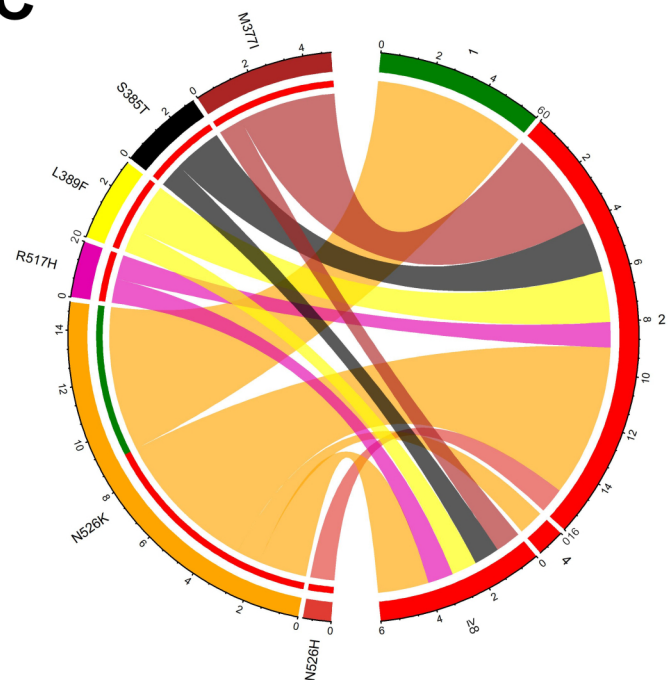**D**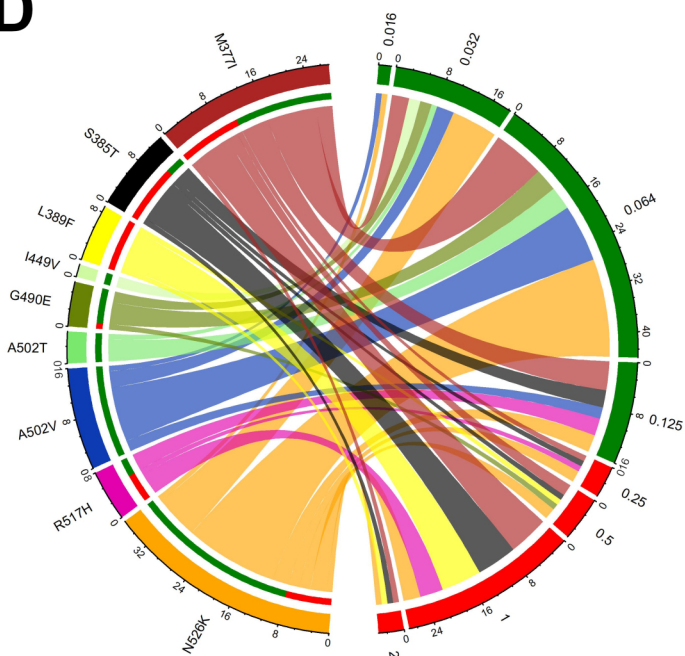**E**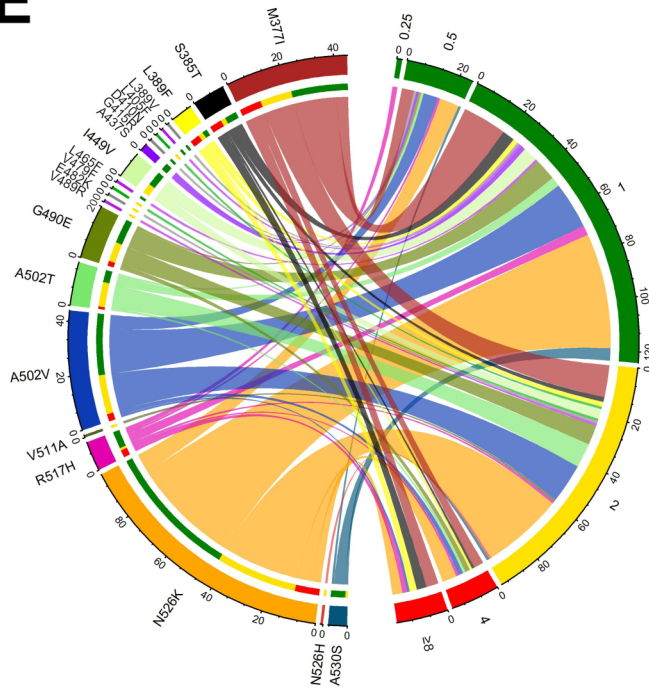**F**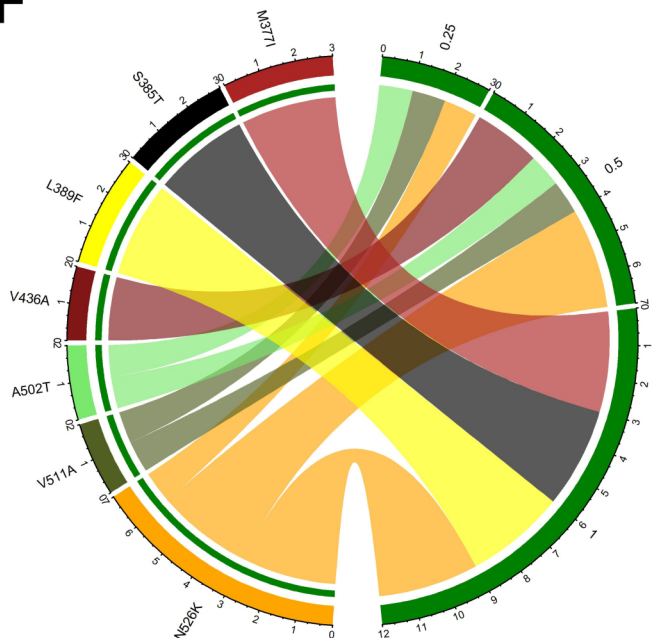

Supplement: Supplementary file 1 — Additional file 1. Supplementary figures. This file contains all Supplementary Figures and the figure captions. Fig. S1 Overview of the cohorts, Fig. S2 Workflow literature review, Fig. S3 Workflow global public cohort, Fig. S4 Workflow GWAS of clinical cohort, Fig. S5 MIC distributions broth microdilution and CLSI clinical breakpoints, Fig. S6 MIC distributions broth microdilution and EUCAST clinical breakpoints, Fig. S7 Ampicillin MICs of group II isolates stratified to methods and cohorts, Fig. S8 Ampicillin MICs of group II sub-groups stratified to different methods, Fig. S9 Circos plots showing the association between PBP3 substitutions and ampicillin/cefotaxime MICs, Fig. S10 Density plot showing the distribution of ftsI mutations, Fig. S11 Distribution of minimum inhibitory concentration based on gradient diffusion strips for the clinical cohort, Fig. S12 Heatmap visualizing the linkage disequilibrium between all amino acid changing variants within the ftsI gene, Fig. S13 Phylogeny of 298 clinical beta-lactamase negative H. influenzae isolates from three European centers (Lübeck, Würzburg and Lisbon), Fig. S14 The haplotype network displaying the 83 combinations of all 44 variants observed in gene ftsI in at least 10 isolates. [file 13073_2024_1406_MOESM1_ESM.zip › Fig S9_Suppl_Figure_circoplots.pdf]
