# Supplementary material for: Revisiting mutational resistance to ampicillin and cefotaxime in Haemophilus influenzae
Source: Genome Med. 2024 Dec 4;16:140. doi: 10.1186/s13073-024-01406-4 (PMC11616347; doi:10.1186/s13073-024-01406-4)
Supplement: Supplementary file 3 — Additional file 3. Supplementary Material. This file contains all Supplementary Material in html format and figure captions. Material S1 Interactive histogram of the distribution of the number of variants per isolate for all 322 beta-lactamase-negative isolates, Material S2 Genome-wide variant-based interactive PCA plot, Material S3: Interactive Manhattan plot of the MIC GWAS, Material S4 Interactive Manhattan plot of the resistance status GWAS, Material S5 Interactive version of Fig. 4C, Material S6 Interactive version of Fig. 4D, Material S7 Interactive heatmap visualizing the linkage disequilibrium between all amino acid changing variants within the ftsI gene. [file 13073_2024_1406_MOESM3_ESM.zip › Material S2_pca.html]

PCA on linear regression dataset
